# The experience of sexuality during the postpartum period: a qualitative study in a city in southern Brazil

**DOI:** 10.1590/1980-220X-REEUSP-2026-0033en

**Published:** 2026-11-09

**Authors:** Viviane Vanderlinde da Silva, Matheus da Cunha Paris, Manuela Beatriz Velho, Luiza Todeschini Vieira, Beatriz de Souza Rodrigues, Laís Antunes Wilhelm

**Affiliations:** 1 Universidade Federal de Santa Catarina, Departamento de Enfermagem, Florianópolis, SC, Brasil.

**Keywords:** Sexuality, Postpartum Period, Nursing, Qualitative Research

## Abstract

**Objective:**

To understand the experience of sexuality during the postpartum period.

**Method:**

This qualitative research was conducted using two distinct yet complementary data collection strategies—semi-structured interviews and a self-administered questionnaire with open-ended questions—, both developed from the same script. The study included 37 people who experienced the postpartum period between the second half of 2021 and the end of 2022 in the state of Santa Catarina. Thematic analysis of the data obtained was adopted.

**Results:**

It became evident that the people interviewed consider sexuality important; however, during the postpartum period they had difficulty engaging in it due to fatigue, lack of libido, pain during intercourse, and other factors related to the postpartum period.

**Conclusion:**

It can be concluded that the topic of sexuality is not regularly addressed during postpartum consultations, with only contraceptive methods and the postpartum period being discussed.

## INTRODUCTION

Parenthood, understood as a process that includes the puerperium, constitutes a unique stage in the lives of people who experience pregnancy and the postpartum period, encompassing individual, family, and social dimensions^(1)^. The puerperium, also referred to as the postpartum period, begins with the expulsion of the fetus and extends until the completion of the involutional processes of pregnancy. It is classified into three phases: immediate (days 1–10), late (days 11–40), and remote (from day 41 onward, with no defined endpoint)^(2)^.

During this period, individuals experiencing the puerperium undergo several physical, emotional, behavioral, and sexual changes. Sexuality extends beyond sexual intercourse and reproduction, encompassing gender identity, sexual orientation, pleasure, beliefs, behaviors, and interpersonal relationships. It is influenced by biological, psychological, social, economic, cultural, historical, and religious factors, constituting an essential aspect of human life^(3)^.

The importance of expanding knowledge about sexuality during the postpartum period should be emphasized, as individuals may experience greater vulnerability because of possible health complications and reduced healthcare support compared with other stages of the pregnancy-puerperal cycle. Evidence indicates that, despite its relevance, sexuality during the puerperium remains underexplored in healthcare services and is frequently neglected or addressed superficially^(4)^.

Furthermore, this topic is often overlooked even during prenatal care, a period in which guidance is predominantly focused on the biological aspects of pregnancy, childbirth, and newborn care, with limited attention to the sexual and relational dimensions of people who experience pregnancy and the puerperium^(5)^. This gap reflects, in part, healthcare professionals’ lack of preparedness to address sexuality comprehensively, whether because of insufficient specific training, the presence of sociocultural taboos, or difficulties in communicating with service users^(6)^.

In this context, nurses play a fundamental role in promoting dialogue about sexuality, contributing to clarifying doubts and fostering a more supportive experience during this period^(7)^. However, the study indicates the need for professional training and the incorporation of systematic educational practices that address sexuality as an essential component of comprehensive healthcare for people who experience pregnancy and the puerperium^(6)^.

Particular emphasis should be given to the Brazilian National Policy on Sexual and Reproductive Rights, established by the Brazilian Ministry of Health in 2005^(8)^, which encourages educational actions in sexual and reproductive health, and to the Sustainable Development Goals of the 2030 Agenda, especially with regard to ensuring universal access to sexual and reproductive healthcare and reproductive rights^(9)^. In this regard, expanding the discussion of sexuality during the puerperium contributes to improving healthcare and strengthening public policies aimed at equity, comprehensive care, and the promotion of the rights of people who experience pregnancy and the puerperium^(10)^.

Despite this, sexuality, particularly during the puerperium, is still insufficiently addressed in healthcare services, which frequently adopt a biologically oriented model centered on reproduction and contraception. This highlights the need for greater professional preparedness and stronger relationships with service users^(10)^.

Given this context, this study aimed to understand the experience of sexuality during the puerperium.

## METHOD

### Study Design

This was a qualitative field study and represents a subanalysis of the larger project entitled “Female sexuality in the postpartum period: women’s experiences”. A qualitative approach was considered necessary to achieve the study objectives because it addresses the values and beliefs of social actors^(11)^. It should also be noted that the study was guided by the COnsolidated criteria for REporting Qualitative research, which provides guidelines for the planning, conduct, and reporting of qualitative research^(12)^.

### Setting

The study was conducted in the municipalities of Greater Florianópolis, in Santa Catarina.

### Population and Selection Criteria

Data were collected using two distinct and complementary strategies—semi-structured interviews and a self-administered questionnaire with open-ended questions—both developed from the same interview guide to ensure thematic consistency across the different modes of data production.

### Data Collection and Sample Definition

Data collection was carried out using two distinct and complementary strategies: semi-structured interviews and a self-administered questionnaire with open-ended questions, both developed from the same questionnaire to ensure thematic coherence between the different modes of data production.

The semi-structured interviews were conducted synchronously and remotely using the Google Meet^®^ platform and were characterized as a dialogic process between the researcher and the participant. This format enabled a deeper exploration of participants’ narratives, the exploration of meanings, and the clarification of emerging interpretations through interaction, in accordance with the assumptions of qualitative research. The interviews lasted approximately 40 minutes, were audiorecorded with participants’ permission, and were subsequently transcribed verbatim for analysis.

Concurrently, a self-administered questionnaire with open-ended questions was made available through the Google Forms^®^ platform. Unlike the interview, the questionnaire did not involve dialogic interaction but rather consisted of a written self-report instrument. This strategy was adopted to broaden the reach of the study and facilitate the participation of individuals who had experienced the puerperium and who expressed embarrassment or reluctance to openly discuss issues related to sexuality in an interactive setting, particularly considering the intimate nature of the topic under investigation. The average completion time for the questionnaire was approximately 20 minutes.

The study was publicized and participants were recruited through messaging applications and social media platforms, including WhatsApp^®^ and Instagram^®^. Snowball sampling was therefore used, as it is recommended for studies involving sensitive topics, hard-to-reach populations, and geographically dispersed contexts^(13)^.

A total of 37 individuals participated in the study, including 14 through synchronous interviews and 23 through the self-administered questionnaire. The number of participants was determined according to the data saturation criterion, considering the recurrence of meanings in the narratives. Data collection took place between the second half of 2021 and the end of 2022.

Before completing the questionnaire or participating in the interview, participants were given access to the Informed Consent Form (ICF) and could either agree or decline to participate. After providing consent, sociodemographic and obstetric information was collected, including age, municipality of residence, marital status, educational level, number of pregnancies and children, self-reported race/ethnicity, religion, and information related to the most recent pregnancy. Following the sociodemographic section, participants were asked the opening question, “What does sexuality mean to you?”, followed by complementary questions regarding their experience of sexuality before, during, and after pregnancy. For the present study, only the data related to the puerperium were analyzed. The data collection instrument was previously tested through a pilot interview, and no modifications were required.

### Data Analysis and Processing

Data analysis followed Minayo’s operative proposal, which encompasses two dimensions: the historical-social and the interpretive. This approach is organized into two operational levels: the historical-social level, which considered the characteristics of the study population, including socioeconomic aspects, and the interpretive level, which sought to identify meanings, logics, and interpretations related to sexuality during the puerperium in participants’ reports. To conduct the second level, it was necessary to organize and classify the data and the responses obtained from the interviews^(14)^.

The first stage consisted of transcribing and rereading the collected material. In the second stage, data were classified through horizontal reading, aimed at identifying initial impressions and central ideas, followed by transversal reading, which enabled a more in-depth understanding of the empirical material. Subsequently, the final analysis was carried out in conjunction with the scientific literature, making it possible to answer the study’s guiding question and achieve the proposed objective^(14)^.

Based on the results, three categories emerged: “Perceptions of changes in sexuality during the puerperium”; “Influence of the mode of birth on the experience of sexuality during the puerperium”; and “Guidance on sexuality during the puerperium”. To ensure participants’ anonymity, the letter “E”, followed by the numerical order corresponding to each participant’s enrollment in the study, was used.

### Ethical Aspects

The macro-project was approved by the Research Ethics Committee of *Universidade Federal de Santa Catarina* on March 29, 2021 (Opinion 4,619,478 and Certificate of Presentation for Ethical Consideration 42605020.0.0000.0121), to which the present study is linked. The ICF was made available before data collection and sent electronically to participants. All ethical principles governing research involving human participants were respected, and all participants signed the ICF.

## RESULTS

Participants’ profile showed that most were between 21 and 40 years of age, with the highest proportions in the 21–25-year (24.3%) and 31–35-year (24.3%) age groups. Concerning place of residence, 62.1% lived in Florianópolis. With respect to gender identity, all participants identified themselves as cisgender women (100%). Most participants were married (56.7%) and had either completed (32.4%) or incomplete (29.7%) higher education. Regarding self-reported race/ethnicity, 62.2% identified as white, 21.6% as mixed-race, 13.5% as black, and 2.7% as Indigenous. Concerning religion, similar proportions of participants identified as Catholic and as having no religion (29.7%).

At the time of the interview, most participants (94.6%) were in the remote puerperium. Regarding the number of pregnancies, 67.6% had experienced one pregnancy, whereas 32.4% had experienced two or more. Concerning the mode of birth, 62.2% had a vaginal birth and 35.1% had a cesarean birth. Among those who had a vaginal birth, 82.6% experienced spontaneous perineal laceration, 13.1% underwent episiotomy, and 4.3% reported no complications.

It was observed that 76.5% of participants reported not having returned to their pre-pregnancy pattern of sexual activity, and 59.5% considered their current sexual life to be worse than before pregnancy.

Data analysis resulted in the emergence of three categories: “Perceptions of changes in sexuality during the puerperium”; “Influence of the mode of birth on the experience of sexuality during the puerperium”; and “Guidance on sexuality during the puerperium”.

### Perceptions of Changes in Sexuality During the Puerperium

Participants described sexuality as a fundamental element of quality of life, associating it with terms such as “connection” (with oneself and with another person), “bond”, “pleasure”, “emotion”, “physical and emotional attraction”, “desire”, “affection”, “sex/sexual intercourse”, and “health”. Regarding its importance, sexuality was considered essential because of its influence on mood, health, and creative energy. It was even perceived as being as important to a relationship as respect and fidelity, constituting a pillar of healthy relationships.

*It affects mood and the willingness to engage in other activities.* (E6)

*To me, sexuality is important for the balance of our body, mind, and health, because when we engage in it, we feel better and healthier.* (E13)

*Important for self-esteem; it relaxes and connects the couple.* (E18)

Some participants reported that sexuality had once been more important in their lives but that their interest in and need for it had decreased because of the multiple demands associated with parenthood, work, household activities, and studies. Fatigue was mentioned as the predominant factor during this period, contributing to the couple’s intimacy being placed in the background. In addition, some participants reported sharing their bed with their baby, suggesting that this practice influenced their sexual life.

*Without motivation, exhaustion takes over. I’m still breastfeeding. And I’m afraid the baby might wake up during the act and kill the mood completely.* (E6)

[My sex life is] *far from being a priority, even though I feel good when I engage in it.* (E8)

*Today I want to take care of my daughter, become financially stable, and study, so I’m so focused on other things that* [sex life] *ends up taking a backseat* [...] *after she was born I didn’t resume that, also because she sleeps in bed with me, I don’t feel comfortable, and I’m the one who takes care of her all the time and I like doing it alone. So, motherhood definitely had a major influence on my sexuality.* (E33)

One participant emphasized that, after childbirth, she began to perceive sexuality as an expression of affection, whereas previously she had understood it predominantly as a physical act.

[...] *after having my daughters, sexuality for me is no longer just about the sexual act; it has become much more about exchanging affection, the love I have to give, and only then do I have and want the sexual act. Before, there was no need for affection or tenderness; it was simply the act itself, and that’s the biggest difference I notice now in the postpartum period.* (E29)

Another participant reported believing that sexuality had become less important in her life, which she associated with the length of her relationship.

*It used to be more important, but now I’m in a period where it’s not as important anymore* [...] *I don’t know if it’s because I had my son or because of the length of the relationship and the time we spent together. I’ve never been with someone for so long. I think that could also be it.* (E24)

Concerning changes in sexuality, some participants reported pain and discomfort during penetration. However, through self-awareness and dialogue with their partners, they came to understand the need for greater stimulation to achieve sexual pleasure during the puerperium. It is noteworthy that this experience was reported even years after childbirth.

*But it was difficult. During those months, I kept thinking the pain was because it was too tight down there, but when I relaxed, I realized it was because I was tense. When I relaxed, the muscle no longer hurt, so I told my husband to stimulate me a lot so I could relax.* (E29)

*I was eager to get back to* it [sexual activity] *soon, until I discovered it hurt a lot. I couldn’t wait for the 40 days* [of the recovery period] *to be over. I kept waiting for the pain to pass, I thought it would go away soon, but it never did in those three years.* (E24)

Participants also reported perceiving “pressure” from both their partners and society to resume sexual activity early. Some mentioned engaging in sexual intercourse to satisfy their partners despite experiencing discomfort. In addition, one participant reported having penetrative sex because she feared muscle atrophy, revealing a lack of knowledge about the functioning of the female body.

*I had absolutely no desire to have sex, but my husband started to feel the need and kept asking, so I even did it to satisfy him, but it was awful, completely uncomfortable* [...] *I don’t feel any desire at all, but he is my daughter’s father.* (E4)

*When I first had sex* [after giving birth], *it was mostly due to pressure... if it were today, I would have waited until I was truly ready. When I was pregnant and said I wanted a natural birth, my father said to me, “You’re going to ruin the man’s little toy”. My father is 56 and holds sexist views. After he said that, I did a lot of thinking, I was in the early stages of pregnancy, and I decided I didn’t want my partner with me during the birth anymore... we get so hung up on what society says about resuming sexual activity quickly, the idea that otherwise, the man won’t be able to handle it and will look elsewhere.* (E31)

Other issues were also mentioned, such as the difficulty of separating sexual life from the demands of parenthood, the influence of breastfeeding, and the perception that partners became less interested in maintaining their sexual life after becoming fathers.

*It’s been a terrible experience; it seems like motherhood destroyed my partner’s libido.* [...] *It seems my partner no longer desires me. On the few occasions we tried, after endless insistence on my part, the baby woke up*. [...] [I feel] *very frustrated.* (E11)

[...] *sometimes we portray the sexual act as something dirty, even knowing that it’s natural and that everyone does it, but that’s something I still need to deconstruct in my mind. I have this duality: I know sex is normal and universal, but my maternal side makes me think about things like my breasts, since she nurses from them. Separating the roles of mother and woman is really difficult.* (E33)

To describe their sexual life after childbirth, participants used terms such as “scarce”, “terrible”, “barely active”, “without libido”, “nonexistent”, and “painful”. However, a minority described it as similar to the pre-pregnancy period, excellent, or active.

When comparing their sexual life during pregnancy with that during the puerperium, most participants (59.5%) described it as worse. The reasons mentioned included lack of availability, breastfeeding, reduced sexual desire, and pain or discomfort during sexual intercourse.

On the other hand, seven participants reported that their sexual life had improved after childbirth, attributing this perception to greater autonomy, empowerment, body self-awareness, and increased sexual pleasure. Other factors mentioned included no longer sharing the bedroom with the baby, the end of breastfeeding, masturbation, and strengthened respect and the marital relationship.

*I think it has changed quite a bit, even in terms of frequency, but I enjoy it more and get more pleasure now, compared to before*. (E25)

[...] *I noticed an improvement in the sexual aspect after the baby left our room, when she started sleeping in her own room; it seems like we’ve gone back to having a life as a couple* [...] *one difference I see is even in relation to the man, that he sees us as women again, not just as mothers.* (E27)

*When I feel the urge, I resort to masturbation, for example. I shut myself off in that moment, sometimes in the shower* [...] *thinking about myself, about feeling pleasure, I feel good. I feel more womanly, more prepared, more involved. I know my body better because I’ve already had a natural childbirth, so everything develops more easily, in a more natural way.* (E30)

Regarding the return to their pre-pregnancy pattern of sexual life, 26 participants (76.5%) reported that they had not resumed that pattern. The reasons cited included reduced sexual desire, overload of activities, greater dedication to childcare, reduced romance, and bodily changes. One participant reported that she was not seeking to return to her previous sexual life because she considered her current circumstances to be different from those before pregnancy.

*It has changed a lot. Even today, four years later, it’s difficult, it seems like I’ve lost the desire to have sex, to have an active sex life*. (E7)

[...] *another important thing is the couple’s priorities, which have changed. We live for the children and end up forgetting a lot about the relationship as a couple. I think this part deserves to be pointed out. There is a lack of romance, a lack of affection, and I think this should be worked on with the couple, who have children and forget to “be a couple” and only think about being parents.* (E28)

*It didn’t happen, and I’m not hoping it will. I’ve already understood that my body is different now, that I’m in a different phase of my life, and I respect that very much. I have very frank conversations with my partner about him not expecting me to be the same person I was two and a half years ago when we were having affairs, because that person no longer exists, even though we have good memories from that time.* (E31)

These findings highlight the relevance of sexuality in participants’ lives, recognizing it as an aspect that contributes to both health and relationship quality. During the puerperium, sexual life tended to be neglected because of factors such as reduced sexual desire, fear, pain, fatigue, and lack of time associated with the demands and responsibilities of this period. Nevertheless, the importance of intimacy within the couple was recognized, with dialogue being highlighted as a fundamental element for coping with the adversities experienced during this stage.

### Influence of the Mode of Birth on the Experience of Sexuality During the Puerperium

The interviews made it possible to identify issues related to puerperal sexuality associated with the mode of birth and its possible repercussions. Many participants reported a negative influence of either vaginal birth or cesarean birth related to pain during sexual intercourse, fear of resuming sexual activity, reduced libido, decreased lubrication, and difficulties recovering from perineal lacerations or episiotomy. Other complications, such as hematomas and hemorrhoids, were also mentioned as factors affecting the sexual experience. In addition, insecurities related to body image emerged, particularly regarding the appearance of the genitalia.

*I had to get stitches and developed hemorrhoids; this had a negative impact on my sexuality.* (E4)

*I had both an episiotomy and a tear, so I experienced a lot of discomfort, and even after a year, I still feel some discomfort.* (E20)

[...] *I didn’t know what it was like down there. I was also ashamed of my body, which was compounded by my fear of getting hurt* [...] *it was shame about that specific area, about the state it was in. I didn’t know what it looked like.* (E37)

Participants expressed dissatisfaction with their sexual life after childbirth, associating it with factors such as pain, reduced lubrication, decreased libido, and bodily changes. In addition, those who had a vaginal birth reported insecurity regarding the appearance of their genitalia, whereas those who underwent cesarean birth mentioned discomfort related to the surgical scar, including altered sensation and concern about possible effects on their relationship.

[The cesarean birth] *makes me think of something aggressive; it limited me for a while. It’s difficult to live with something that took so long to heal. I still can’t feel that area because I lost sensation around the scar. It feels like a wound that wasn’t only physical; I also felt down and upset, and it affected me emotionally as well.* (E32)

### Guidance on Sexuality During the Puerperium

In relation to guidance on sexuality after childbirth, this topic was found not to be addressed systematically during follow-up consultations or within the context of parenthood. Approximately 43.2% of participants reported not having received any professional guidance regarding the resumption of sexual activity. Faced with this gap in care, many individuals who had experienced the puerperium reported seeking information from the internet, friends, and family members to clarify their doubts. These findings suggest weaknesses in addressing sexuality during puerperal care, highlighting the need for greater incorporation of this topic into health education activities and postpartum follow-up.

*I didn’t receive* [guidance]. *The knowledge I had about this stems from the fact that I studied it.* (E9)

*I had questions, and I clarified them through the GAPP* [Postpartum Support Group] *and virtual meetings on the subject.* (E18)

The findings showed that healthcare professionals tend to limit their guidance to contraceptive methods, with less attention given to broader aspects of sexuality after childbirth. Participants emphasized the importance of an approach that normalizes the changes experienced after childbirth and the need to establish rapport and a trusting relationship with healthcare professionals. Only 43.2% of participants reported having received any guidance, which, in most cases, was limited to the recommendation to observe a 40-day postpartum recovery period.

*Actually, they only recommended I take birth control pills, that’s all. They told me this after the birth, right after he was born, the nurse advised me.* (E34)

*I was only instructed to wait the 40 days, nothing more than that. The obstetrician gave me this advice at the time of hospital discharge. I wasn’t given this advice during prenatal care or at other times, for example.* (E27)

Some participants reported having doubts about resuming sexual activity but chose not to seek clarification, instead waiting until they felt more comfortable gradually resuming sexual activity. One participant reported not following the traditional 40-day postpartum recovery period, choosing instead to resume her sexual life when she felt physically and emotionally ready.

*I had questions. I wondered whether there was an appropriate time to resume sexual activity and whether, because I had a cesarean birth, my body would be ready after a certain period. Those were my main questions. I didn’t seek answers; I waited for my body. When I felt safe, when I no longer felt discomfort when we tried, it eventually happened. But I didn’t look for information or seek medical advice; I simply observed my body and how it responded.* (E26)

*I had no doubts, and I didn’t even follow the advice to wait the 40 days. For me, when a woman feels good and is comfortable with it, she can resume her sex life whenever she feels like it.* (E27)

The findings of this study show that sexuality during the puerperium is still seldom addressed during Primary Health Care consultations and within the context of parenthood, with guidance predominantly focused on contraceptive methods. Weaknesses were also observed in counseling regarding the postpartum recovery period and the resumption of sexual activity. In this context, many participants sought information from informal sources, such as the internet, friends, and family members, which may contribute to the persistence of doubts and insecurities. These findings reinforce the need to broaden the discussion of sexuality during puerperal care by incorporating it systematically into health education activities and postpartum care.

## DISCUSSION

### Perceptions of Changes in Sexuality During the Puerperium

Participants recognized sexuality during the puerperium as an aspect that extends beyond sexual intercourse and reproduction, considering it essential throughout life, regardless of age. However, a contradiction was observed between this understanding and actual practice, as many participants still restricted sexuality to penetrative intercourse^(15)^.

Within the puerperal context, female sexual dysfunction deserves particular attention, as it encompasses changes in sexual desire, arousal, orgasm, and sexual pain^(16)^. The hormonal, physical, and psychoemotional changes occurring during this period may affect sexual life, and sexual dysfunction is frequently associated with factors such as episiorrhaphy, perineal lacerations, and postpartum depression^(17)^. These aspects are consistent with participants’ reports, which predominantly described sexual life negatively. Reduced libido was also associated with breastfeeding, an activity frequently described as exhausting and one that may cause discomfort when associated with sexual stimulation. Considering that the breasts have erotic significance, their primarily maternal function during this period may influence the experience of sexuality. In this context, it is important for puerperal individuals to distinguish between maternal and sexual stimulation and to deconstruct beliefs that associate sexuality with impurity^(18)^.

Sociocultural factors, including socialization experiences, culture, and religion, influence sexuality throughout life^(19)^. During the puerperium, childcare demands may reduce opportunities for privacy and intimacy between partners^(20)^. At the same time, some individuals attribute negative meanings to sexuality and report difficulties integrating different identity dimensions related to caregiving, the body, and sexual life. Conversely, social and partner pressure to resume sexual activity early was also perceived. This context reveals tensions and contradictions associated with the social construction of gender, as society has historically established norms and standards that may reinforce inequalities and conflicts in the experience of sexuality^(21)^.

Furthermore, the findings showing that some participants engaged in sexual intercourse to satisfy their partners or avoid infidelity can be understood in light of gender and power relations, in which social norms continue to influence the experience of sexuality during the puerperium. A recent study indicates that the resumption of sexual activity after childbirth is shaped by negotiation processes within intimate relationships. Within these processes, sexual activity may be socially perceived as an expectation of the relationship, with possible implications for the sexual autonomy and sexual and emotional health of those involved^(22)^.

Partners play an important role in providing emotional and practical support, although this support is not always effective and may result in communication difficulties and conflicts. Adaptation to parenthood imposes challenges on both the couple’s relationship and their sexual life. Therefore, the importance of family and professional support throughout the pregnancy-puerperal cycle should be emphasized, including educational actions addressing the physiological and emotional changes of the puerperium and their effects on sexuality^(23)^.

In addition, it is important to consider that the time elapsed between the puerperium and data collection may have influenced the perceptions reported by participants. Although recalling experiences allows individuals to elaborate on and reinterpret lived events, it may also be subject to recall bias, since retrospective reports involve processes of reconstruction and interpretation over time, which may attenuate or intensify lived experiences. Therefore, the reports may reflect not only what was experienced at that time but also interpretations developed subsequently, which should be taken into account when interpreting the findings^(24)^.

### Influence of the Mode of Birth on the Experience of Sexuality During the Puerperium

Several factors influence the time to resume sexual activity during the puerperium, including maternal comorbidities, fear of a subsequent pregnancy, fear of pain, the degree of perineal injury, and breastfeeding^(25)^. Among participants, the perception emerged that the mode of birth may affect sexuality, particularly because of lacerations or episiotomies, which may lead to dyspareunia, fear, reduced libido, and decreased lubrication. In addition, insecurities related to the cesarean scar and changes in the genitalia were reported.

Vaginal birth may result in genital changes, such as perineal trauma and dyspareunia, although it offers benefits for maternal and neonatal health, with possible complications that may be managed through physical therapy. Cesarean birth, in turn, may cause scar discomfort and affect sexual function, although there is no direct association between the mode of birth and sexual function^(26)^. Evidence indicates that individuals who undergo cesarean birth or operative vaginal birth with laceration or episiotomy may take longer to resume sexual activity, whereas having a stable partner is a facilitating factor^(27)^.

In this context, the importance of the childbirth humanization movement should be emphasized, as it seeks to reduce medicalization and obstetric violence while promoting women’s autonomy and recognition of their rights. With regard to sexuality, interventions such as episiotomy may result in perineal trauma, dyspareunia, and possible sexual dysfunctions, whereas lacerations may occur physiologically during labor. Both may be prevented or minimized through pelvic floor physical therapy and perineal stretching techniques, although these practices remain underutilized despite being minimally invasive^(28)^.

The reports also revealed dissatisfaction with body image after childbirth, related both to changes resulting from vaginal birth and to the cesarean scar. Although expected, these aesthetic bodily changes may lead to frustration and affect the self-image and sexual life of individuals who have experienced the puerperium^(29)^. Thus, different modes of birth may influence the sexual experience during the puerperium, reinforcing the importance of guidance, opportunities for dialogue on the topic, and access to reliable information about the body, sexuality, and care during this period.

In this regard, body image is an important psychological determinant of sexuality and may precede and influence sexual desire. Recent evidence indicates that a negative perception of one’s body during the postpartum period is associated with reduced self-esteem, decreased sexual satisfaction, and greater emotional vulnerability, reinforcing the need for approaches that consider women’s subjectivity during this period^(30)^.

### Guidance on Sexuality During the Puerperium

The focus of postpartum consultations remains centered on conception, with limited inclusion of dialogue with partners^(31)^. Considering the five dimensions of individual health, it is essential to address sexuality and its impact on the quality of life of puerperal individuals and their partners^(32)^. The interviews revealed a lack of professional information validating the experiences commonly encountered during this period, reinforcing the need for qualified guidance.

Additionally, it is important to consider that participants’ profile, consisting predominantly of white women with higher educational attainment, may influence access to information and healthcare. The literature indicates that social markers such as race, social class, and education, understood from the perspective of intersectionality, affect access to healthcare services and the quality of care received. Women in more vulnerable contexts may therefore face even greater barriers to accessing qualified guidance^(33)^.

Given the influence of the internet on communication and health education, it is frequently used to clarify doubts. However, the information obtained may generate insecurity and negatively affect the experience of sexuality. Issues related to sexual activity continue to raise questions among puerperal individuals and are associated with embarrassment and reluctance to discuss the topic with healthcare professionals^(34)^.

The importance of providing information to couples and training professionals to address sexuality during the puerperium is well recognized. In Brazil, the Ministry of Health provides the Primary Care Notebooks on Sexual and Reproductive Health, which guide qualified practices in this field^(35)^. The Permission, Limited Information, Specific Suggestions, Intensive Therapy model is highlighted as a consultation model that can be applied by nurses in the management of sexual dysfunctions. The recommendations emphasize consideration of the sociocultural context, the provision of emotional and psychological support, and the adoption of non-pharmacological approaches, including high-quality sexual education. Furthermore, counseling should begin during prenatal care by identifying sexual dysfunctions and providing guidance on physiological changes to prepare couples for the pregnancy-puerperal cycle^(36)^.

The findings suggest that healthcare professionals, including nurses, still neglect the topic of sexuality, as reflected by the limited number of studies on therapeutic interventions. Discussing this topic may promote more open communication among pregnant women, puerperal individuals, and healthcare professionals, as well as encourage future research. Therefore, it is essential to train nurses, in particular, since sexuality is a fundamental component of healthcare. Healthcare teams should provide effective care beginning in prenatal care, taking each individual’s specific characteristics into account in order to address difficulties related to sexuality^(37)^.

Although the number of participants was appropriate for the study proposal, it does not allow generalization of the findings, considering the size of the population under investigation. Nevertheless, qualitative research seeks to understand the phenomenon through participants’ narratives and individual experiences. Therefore, further studies are recommended using active recruitment strategies and broader dissemination combined with snowball sampling to increase the representativeness of puerperal individuals.

Among the study limitations, the time elapsed between the puerperal experience, data collection (2021-2022), and publication should first be highlighted, as it may have influenced participants’ reports through processes of recall, elaboration, and reinterpretation of their lived experiences. Although memory allows more mature reflection on lived experiences, retrospective reports are subject to recall bias and may attenuate or intensify certain perceptions.

Another limitation concerns participants’ profile, which consisted predominantly of white women with higher educational attainment and living in urban settings. These social markers of race, class, and gender may influence access to information, healthcare services, and care opportunities, limiting the transferability of the findings to women living in contexts of greater social, racial, and economic vulnerability.

Furthermore, although the sample of 37 participants allowed the identification of recurring themes and the achievement of data saturation, it represents a relatively large number for qualitative studies requiring greater analytical depth at the individual level, which may have limited a more detailed exploration of each narrative. This limitation is further compounded by the combined use of dialogic interviews and self-administered questionnaires, since the latter format restricts the in-depth exploration of emerging meanings compared with synchronous interviews.

As a contribution to nursing, this study is expected to encourage professional development and broaden the discussion of female sexuality from prenatal care through the puerperium, fostering dialogue on this topic across the different settings in which nurses practice. In addition, the findings may support future research and contribute to improving continuing health education initiatives.

## CONCLUSION

This study explored the experience of sexuality during the puerperium and demonstrated that, for many individuals, experiencing sexuality in a satisfying way during this period is challenging. Although participants recognized its importance for health, self-esteem, and the maintenance of healthy relationships, the demands of parenthood frequently reduced the time available for intimacy, making dialogue between partners essential for preserving their relationship.

Biological factors, including pain during sexual intercourse, reduced libido, the effects of breastfeeding, and dissatisfaction with body image, were identified as barriers to returning to the pre-pregnancy pattern of sexual activity. Furthermore, both vaginal birth and cesarean birth were associated with discomforts that may affect sexuality. Weaknesses were also identified in healthcare professionals’ approach to sexuality throughout the pregnancy-puerperal cycle, with guidance predominantly limited to contraceptive methods and the postpartum recovery period.

This study reinforces the need to broaden the approach to sexuality in healthcare, particularly in nursing, from undergraduate education to clinical practice, promoting health education and qualified support for individuals experiencing the puerperium.

## Data Availability

The entire dataset supporting the results of this study is available at: https://repositorio.ufsc.br/handle/123456789/248224.
