# Supplementary material for: The experience of sexuality during the postpartum period: a qualitative study in a city in southern Brazil
Source: Rev Esc Enferm USP. 2026 Nov 9;60:e20260033. doi: 10.1590/1980-220X-REEUSP-2026-0033en (PMC13754459; doi:10.1590/1980-220X-REEUSP-2026-0033en)
Supplement: Translation

# Translation: Vivência da sexualidade no puerpério: estudo qualitativo em uma cidade do Sul do Brasil

**DOI:** 10.1590/1980-220X-REEUSP-2026-0033pt

**Authors:** Viviane Vanderlinde da Silva, Matheus da Cunha Paris, Manuela Beatriz Velho, Luiza Todeschini Vieira, Beatriz de Souza Rodrigues, Laís Antunes Wilhelm

**Affiliations:** 1 Universidade Federal de Santa Catarina, Departamento de Enfermagem, Florianópolis, SC, Brasil.

**Keywords:** Sexualidade, Período Pós-Parto, Enfermagem, Pesquisa Qualitativa

## Abstract

**Objetivo:**

Compreender a vivência da sexualidade no puerpério.

**Método:**

Pesquisa de abordagem qualitativa, realizada por meio de duas estratégias de coleta de dados distintas e complementares — entrevistas semiestruturadas e formulário autoaplicável com questões abertas —, ambas elaboradas a partir de um mesmo roteiro. Participaram do estudo 37 pessoas que vivenciaram o puerpério no estado de Santa Catarina entre o segundo semestre de 2021 e o final de 2022. Adotou-se a análise temática dos dados obtidos.

**Resultados:**

Evidenciou-se que as pessoas entrevistadas consideram a sexualidade importante, mas, no puerpério, elas tiveram dificuldade em exercer por conta do cansaço, da falta de libido, da dor durante a relação, entre outros fatores relacionados ao pós-parto.

**Conclusão:**

Conclui-se que o tema da sexualidade não é regularmente abordado durante a consulta puerperal, sendo abordados apenas métodos contraceptivos e tempo de resguardo.

## INTRODUÇÃO

A parentalidade, compreendida como um processo que inclui o puerpério, constitui uma fase singular na vida das pessoas que gestam e vivenciam esse período, abrangendo dimensões individuais, familiares e sociais^(1)^. O puerpério, também denominado pós-parto, inicia-se com a saída do feto e se estende até o término dos processos involutivos da gestação, sendo classificado em três fases: imediata (1º ao 10º dia), tardia (11º ao 40º dia) e remota (a partir do 41º dia, sem término definido)^(2)^.

Nesse período, a pessoa que vivencia o puerpério experimenta diversas transformações físicas, emocionais, comportamentais e sexuais. A sexualidade ultrapassa o ato sexual e a reprodução, englobando identidade de gênero, orientação sexual, prazer, crenças, comportamentos e relações interpessoais. Ela é influenciada por fatores biológicos, psicológicos, sociais, econômicos, culturais, históricos e religiosos, configurando-se como um aspecto essencial da vida humana^(3)^.

Destaca-se a importância de ampliar o conhecimento sobre a sexualidade no pós-parto, fase em que as pessoas podem apresentar maior vulnerabilidade devido a possíveis complicações de saúde e à menor assistência em comparação a outras etapas do ciclo gravídico-puerperal. Evidências apontam que, apesar da relevância do tema, a sexualidade no puerpério permanece pouco explorada nos serviços de saúde, sendo frequentemente negligenciada e abordada de forma superficial^(4)^.

Além disso, observa-se que a temática é, muitas vezes, invisibilizada ainda no pré-natal, período em que predominam orientações voltadas aos aspectos biológicos da gestação, do parto e dos cuidados com o recém-nascido, com limitada abordagem das dimensões sexuais e relacionais das pessoas que gestam e vivenciam o puerpério^(5)^. Tal lacuna reflete, em parte, o despreparo dos profissionais de saúde para abordar a sexualidade de forma integral, seja pela insuficiência de formação específica, pela presença de tabus socioculturais ou por dificuldades na comunicação com as usuárias^(6)^.

Nesse contexto, o enfermeiro possui papel fundamental ao promover o diálogo sobre a sexualidade, contribuindo para o esclarecimento de dúvidas e para uma vivência mais acolhedora desse período^(7)^. No entanto, o estudo indica a necessidade de qualificação profissional e da incorporação de práticas educativas sistematizadas que contemplem a sexualidade como um componente essencial da assistência integral à saúde das pessoas que gestam e vivenciam o puerpério^(6)^.

Destacam-se a Política Nacional de Direitos Sexuais e Reprodutivos, instituída pelo Ministério da Saúde em 2005^(8)^, que incentiva ações educativas em saúde sexual e reprodutiva, e os Objetivos de Desenvolvimento Sustentável da Agenda 2030, especialmente no que se refere à garantia do acesso universal à saúde sexual e reprodutiva e aos direitos reprodutivos^(9)^. Nesse sentido, a ampliação da abordagem da sexualidade no puerpério contribui para a qualificação da assistência à saúde e para o fortalecimento de políticas públicas voltadas à equidade, à integralidade do cuidado e à promoção dos direitos das pessoas que gestam e vivenciam o puerpério^(10)^.

Apesar disso, a sexualidade, especialmente no puerpério, ainda é pouco abordada nos serviços de saúde, que frequentemente adotam um modelo biologicista centrado na reprodução e na contracepção. Isso evidencia a necessidade de maior preparo dos profissionais e de fortalecimento do vínculo com as usuárias^(10)^.

Diante desse contexto, este estudo teve como objetivo compreender a vivência da sexualidade no puerpério.

## MÉTODO

### Desenho do Estudo

Trata-se de um pesquisa de campo com abordagem qualitativa, sendo um recorte do macroprojeto intitulado “Sexualidade feminina no pós-parto: vivências de mulheres”. Entende-se como necessária a abordagem qualitativa para atender aos objetivos do estudo, pois tratam-se de valores e crenças dos atores sociais^(11)^. Destaca-se, ainda, que a pesquisa foi guiada pelo *COnsolidated criteria for REporting Qualitative research*, que fornece diretrizes para o planejamento, a execução e a elaboração de relatórios de pesquisas qualitativas^(12)^.

### Local

O estudo foi realizado nos municípios da Grande Florianópolis, em Santa Catarina.

### População e Critérios de Seleção

A população do estudo foi composta por pessoas que vivenciaram o puerpério a partir de 2017, maiores de 18 anos, com vida sexual ativa, com o intuito de assegurar maior fidedignidade das recordações. Foram excluídas do estudo as pessoas que apresentaram gestação de alto risco ou que vivenciaram a gestação na adolescência, considerando as possíveis particularidades dessas experiências.

### Coleta de Dados e Definição da Amostra

A coleta de dados ocorreu por meio de duas estratégias distintas e complementares — entrevistas semiestruturadas e formulário autoaplicável com questões abertas —, ambos elaborados a partir do mesmo roteiro de perguntas, de modo a garantir coerência temática entre os diferentes modos de produção dos dados.

As entrevistas semiestruturadas foram realizadas de forma síncrona e remota por meio da plataforma *Google Meet* ^®^, caracterizando-se como um processo dialógico entre pesquisadora e participante. Esse formato possibilitou um maior aprofundamento das narrativas, a exploração de significados e o esclarecimento de sentidos emergentes a partir da interação, conforme os pressupostos da pesquisa qualitativa. As entrevistas, que tiveram duração média de 40 minutos, foram gravadas com a autorização das participantes e, posteriormente, transcritas na íntegra para análise.

Paralelamente, utilizou-se um formulário autoaplicável com questões abertas, disponibilizado por meio da plataforma *Google Forms* ^®^. Diferentemente da entrevista, o formulário não pressupõe interação dialógica, constituindo-se em um instrumento escrito de autorrelato. A adoção dessa estratégia teve como objetivo ampliar o alcance da pesquisa e favorecer a participação de pessoas que vivenciaram o puerpério e que manifestavam receio ou constrangimento em abordar abertamente questões relacionadas à sexualidade em um contexto interativo, sobretudo considerando o caráter íntimo do tema investigado. O tempo médio de preenchimento do formulário foi de aproximadamente 20 minutos.

A divulgação do estudo e o recrutamento das participantes ocorreram por meio de aplicativos de mensagens e redes sociais, como o *WhatsApp* ^®^ e o *Instagram* ^®^. Utilizou-se, assim, a técnica de amostragem em bola de neve, indicada para pesquisas que envolvem temáticas sensíveis e populações de difícil acesso, bem como para contextos de ampla dispersão geográfica^(13)^.

Participaram do estudo 37 pessoas que vivenciaram a situação, sendo 14 por meio de entrevistas síncronas e 23 por meio de um formulário autoaplicável. O número de participantes foi definido a partir do critério de saturação dos dados, considerando a recorrência de sentidos nas narrativas. A coleta foi realizada entre o segundo semestre de 2021 e o final de 2022.

Ao acessarem o formulário ou antes da realização da entrevista, as participantes tiveram acesso ao Termo de Consentimento Livre e Esclarecido (TCLE), podendo aceitar ou recusar sua participação. Após a aceitação, foram coletadas informações sociodemográficas e obstétricas, como idade, município de residência, estado civil, escolaridade, número de gestações e filhos, cor/raça autodeclarada, religião e dados referentes à última gestação. Após a etapa sociodemográfica, foi apresentada a questão disparadora “O que significa sexualidade para você?”, seguida de questões complementares relacionadas à vivência da sexualidade antes, durante e após a gestação. Para este estudo, utilizou-se o recorte referente ao período puerperal. O instrumento de coleta foi previamente testado por meio de entrevista piloto, não havendo a necessidade de ajustes.

### Análise e Tratamento de Dados

A análise de dados foi realizada conforme a proposta operativa de Minayo, que contempla dois aspectos: o histórico-social e o interpretativo. Essa abordagem se organiza em dois níveis operacionais: o histórico-social, que considerou as características do grupo pesquisado, incluindo aspectos socioeconômicos, e o interpretativo, que buscou identificar, nos relatos, sentidos, lógicas e interpretações relacionados à sexualidade no puerpério. Para a realização do segundo nível, foi necessário proceder à ordenação e à classificação dos dados e das respostas obtidas nas entrevistas^(14)^.

A primeira etapa consistiu na transcrição e releitura do material obtido. Na segunda etapa, os dados foram classificados por meio de leitura horizontal, destinada à identificação das impressões iniciais e das ideias centrais, seguida de leitura transversal, que possibilitou o aprofundamento e a compreensão mais detalhada do material empírico. Posteriormente, realizou-se a análise final, articulada à literatura científica, permitindo responder à questão norteadora do estudo e alcançar o objetivo proposto^(14)^.

A partir dos resultados, emergiram três categorias distintas: “Percepções sobre as alterações na sexualidade durante o puerpério”; “Influência dos tipos de parto na vivência da sexualidade no puerpério”; e “Orientações sobre a sexualidade no puerpério”. Para garantir o anonimato das participantes, utilizou-se a letra “E”, seguida da ordem numérica correspondente à participação na pesquisa.

### Aspectos Éticos

O macroprojeto foi aprovado pelo Comitê de Ética em Pesquisa da Universidade Federal de Santa Catarina em 29 de março de 2021 (Parecer nº 4.619.478 e Certificado de Apresentação para Apreciação Ética nº 42605020.0.0000.0121), ao qual o presente estudo está vinculado. O TCLE foi disponibilizado previamente à coleta de dados e enviado às participantes por meio eletrônico. Todos os preceitos éticos para pesquisas com seres humanos foram respeitados, e todas as participantes assinaram o TCLE.

## RESULTADOS

O perfil das participantes demonstra que a maioria tinha entre 21 e 40 anos, com a predominância das faixas de 21 a 25 anos (24,3%) e de 31 a 35 anos (24,3%). Quanto ao local de residência, 62,1% residiam em Florianópolis. Em relação à identidade de gênero, todas se identificaram como mulheres cisgêneras (100%). Predominaram participantes casadas (56,7%), com ensino superior completo (32,4%) ou incompleto (29,7%). Quanto à raça/cor autodeclarada, 62,2% eram brancas, 21,6%, pardas, 13,5%, pretas, e 2,7%, indígenas. Em relação à religião, observou-se uma proporção semelhante entre católicas e sem religião (29,7%).

No momento da entrevista, a maioria (94,6%) se encontrava no puerpério remoto. Quanto ao número de gestações, 67,6% tiveram uma gestação, e 32,4% tiveram duas ou mais. Em relação ao tipo de parto, 62,2% tiveram parto vaginal, e 35,1%, cesariana. Entre os partos vaginais, 82,6% apresentaram laceração espontânea, 13,1%, episiotomia, e 4,3% não relataram intercorrências.

Observou-se que 76,5% das participantes referiram não ter retornado ao padrão sexual anterior à gestação, e 59,5% consideraram a vida sexual atual pior em comparação ao período pré-gestacional.

Da análise dos dados, emergiram três categorias: “Percepções sobre as alterações na sexualidade durante o puerpério”; “Influência dos tipos de parto na vivência da sexualidade no puerpério”; e “Orientações sobre a sexualidade no puerpério”.

### Percepções Sobre as Alterações na Sexualidade Durante o Puerpério

As participantes descreveram a sexualidade como um elemento fundamental para a qualidade de vida, sendo associada a termos como “conexão” (consigo e com o outro), “aliança”, “prazer”, “emoção”, “atração física e afetiva”, “desejo”, “carinho”, “sexo/ato sexual” e “saúde”. Quanto à sua importância, a sexualidade foi considerada essencial, com influência sobre o humor, a saúde e a energia criativa, sendo percebida, inclusive, como tão relevante para o relacionamento quanto o respeito e a fidelidade, configurando-se como um pilar para relações saudáveis.

*Afeta o humor e a disposição para outras atividades.* (E6)

*Para mim a sexualidade é importante para o equilíbrio do nosso corpo, mente e saúde, pois, quando praticamos, nos sentimos melhores e mais saudáveis.* (E13)

*Importante para autoestima; relaxa, conecta o casal.* (E18)

Algumas participantes relataram que a sexualidade já foi mais relevante em suas vidas, porém referiram redução do interesse e da necessidade, atribuída às múltiplas demandas relacionadas à parentalidade, ao trabalho, às atividades domésticas e aos estudos. O cansaço foi mencionado como fator predominante nessa fase, contribuindo para que a intimidade do casal fosse colocada em segundo plano. Além disso, algumas participantes relataram compartilhar a cama com o bebê, sugerindo influência dessa prática na vida sexual.

*Sem vontade... cansaço domina. Ainda estou amamentando. E tenho medo de, durante o ato, bebê acordar e acabar todo clima.* (E6)

[A vida sexual está] *longe de ser uma prioridade, apesar de me sentir bem quando pratico.* (E8)

*Hoje quero cuidar da minha filha, me estabilizar financeiramente e estudar, então estou tão focada em outras coisas que isso* [vida sexual] *acaba ficando pra trás* [...] *depois que ela nasceu, eu não retomei mais isso, até porque ela dorme comigo na cama. Eu não me sinto bem, e sou eu que cuido dela o tempo todo e gosto de fazer isso sozinha. Então, com certeza, a maternidade influenciou muito na minha sexualidade.* (E33)

Uma participante destacou que, após o parto, passou a perceber a sexualidade como uma expressão de carinho, enquanto anteriormente a compreendia predominantemente como um ato físico.

[...] *depois que tive minhas filhas, a sexualidade para mim não é mais só o ato sexual, virou muito mais uma troca de carinhos, o afeto que tenho para dar e só aí ter e querer o ato sexual. Antes não precisava de carinho e afeto, era simplesmente o ato, e essa é a maior diferença que eu percebo agora no pós-parto.* (E29)

Outra participante relatou acreditar que a sexualidade deixou de ser tão importante em sua vida, o que foi associado ao tempo de relacionamento.

*Já teve mais; agora eu estou num período que já não é mais tão importante* [...] *não sei se é porque eu tive meu filho ou pelo tempo de relacionamento e de convivência. Nunca estive tanto tempo com uma pessoa... acho que também pode ser isso.* (E24)

Em relação às alterações na sexualidade, algumas relataram dor e desconforto durante a penetração. Entretanto, por meio do autoconhecimento e do diálogo com o parceiro, compreenderam a necessidade de maior estímulo para alcançar o prazer sexual no puerpério. Destaca-se que essa experiência foi relatada mesmo anos após o parto.

*Mas foi difícil. Nesses meses, eu fui achando que a dor era porque ela tava apertadinha, aí quando relaxei, percebi que era porque eu estava tensa. Quando eu relaxava, não doía mais o músculo, aí eu dizia pro meu marido me estimular bastante para eu relaxar.* (E29)

*Eu estava com vontade de voltar logo* [à atividade sexual], *até descobrir que doía muito. Não via a hora de chegar os 40 dias* [de resguardo]. *Fiquei esperando a dor passar. Achei que logo passaria, mas nunca passou nesses três anos.* (E24)

As entrevistadas também relataram a percepção de “pressão” por parte dos parceiros e da sociedade para o retorno precoce à atividade sexual. Algumas mencionaram manter relações sexuais com o objetivo de agradar o parceiro, mesmo na presença de desconforto. Além disso, uma participante relatou ter realizado sexo penetrativo por receio de atrofia muscular, evidenciando desconhecimento acerca do funcionamento do corpo feminino.

*Eu não tinha vontade alguma de fazer sexo e meu marido começou a sentir necessidade e pedia, e eu até fazia para satisfazê-lo, mas era muito horrível, totalmente desconfortável* [...] *não sinto vontade alguma, mas ele é o pai da minha filha.* (E4)

*Quando eu tive minha primeira atividade sexual* [após o parto]*, foi mais pela pressão* [...] *se fosse hoje, eu teria esperado mais o meu momento mesmo. Quando eu estava grávida, eu escutei do meu pai a seguinte frase quando disse que queria um parto normal: “Vai estragar todo o brinquedinho do homem”. Meu pai de 56 anos, machista. E depois que ele falou isso, eu fiquei muito reflexiva, pois estava no início da gestação, e eu não quis mais meu companheiro comigo no parto* [...] *a gente se prende muito ao que a sociedade diz sobre voltar logo para atividade sexual, porque senão o homem não vai aguentar, que vai procurar fora de casa.* (E31)

Outras questões também foram mencionadas, como a dificuldade de dissociar a vida sexual das demandas da parentalidade, a influência da amamentação e a percepção de menor interesse dos parceiros na manutenção da vida sexual após o envolvimento com a paternidade.

*Está sendo uma experiência péssima, parece que a maternidade destruiu a libido do meu companheiro.* [...] *parece que meu companheiro não sente mais desejo por mim. As poucas vezes que tentamos, após infinitas insistências de minha parte, o bebê acorda* [...] [me sinto] *muito frustrada.* (E11)

[...] *às vezes, a gente coloca o ato sexual como algo sujo, mesmo sabendo que é algo natural e que todo mundo faz, mas isso eu ainda tenho que desconstruir na minha cabeça. Tenho essa dualidade. Eu sei que o ato sexual é algo normal e que todo mundo faz, mas esse meu lado maternal me faz pensar até em coisas como, por exemplo, meus seios, já que ela mama nos meus seios. Essa questão de separar mãe e mulher é muito difícil.* (E33)

Para descrever a vida sexual no pós-parto, as participantes utilizaram termos como “escassa”, “péssima”, “pouco ativa”, “sem libido”, “inexistente” e “dolorosa”. Contudo, uma minoria a descreveu como semelhante ao período anterior, ótima ou ativa.

Ao comparar a vida sexual durante a gestação e no puerpério, a maioria das participantes (59,5%) a classificou como pior. Entre os motivos citados, destacam-se a falta de disponibilidade, a amamentação, a redução do desejo e a presença de dor ou desconforto durante a relação sexual.

Por outro lado, sete participantes relataram melhora da vida sexual no pós-parto, associando essa percepção à maior autonomia, ao empoderamento, ao autoconhecimento corporal e ao aumento do prazer. Também foram mencionados fatores como o término do compartilhamento do quarto com o bebê, o fim da amamentação, a prática de masturbação e o fortalecimento do respeito e da relação conjugal.

*Acho que mudou bastante, até com relação a frequência, mas gosto mais e tenho mais prazer agora, em comparação com antes.* (E25)

[...] *eu percebi melhora da questão sexual depois que a bebê saiu do nosso quarto, quando ela passou a dormir no quarto dela; parece que voltamos a ter uma vida como um casal* [...] *uma diferença que vejo é até com relação ao homem, de ele nos ver como mulher novamente, não só como mãe.* (E27)

*Quando eu sinto vontade, eu apelo pra masturbação, por exemplo. Eu me fecho nesse momento, às vezes, num banho* [...] *pensando em mim, em eu sentir prazer, eu me sinto bem. Me sinto mais mulher, mais preparada, mais envolvida. Conheço melhor o meu corpo por já ter tido um parto normal, então tudo se desenvolve mais fácil, de maneira mais natural.* (E30)

Quanto ao retorno ao padrão de vida sexual anterior à gestação, 26 participantes (76,5%) relataram não ter retomado esse padrão. Entre os motivos apontados, destacam-se a redução do desejo, a sobrecarga de atividades, a maior dedicação aos cuidados com o filho, a diminuição do romantismo e as mudanças corporais. Uma participante relatou não buscar esse retorno, considerando que as circunstâncias atuais são diferentes do período anterior à gestação.

*Mudou bastante. Até hoje, quatro anos depois, tá difícil, parece que perdi a vontade de fazer sexo, de ter uma vida sexual ativa.* (E7)

[...] *outra coisa importante é a prioridade do casal, que mudou. A gente vive pelos filhos e acaba esquecendo muito da relação como casal. Acho que essa parte merece ser apontada. Falta romantismo, falta carinho, e acho que isso deveria ser trabalhado com o casal, que tem filhos e esquece de “ser casal” e só pensa em ser pai e mãe.* (E28)

*Não aconteceu, e eu também não busco que aconteça. Já entendi que meu corpo é outro, que o momento da minha vida é outro, e eu respeito muito isso. Tenho essa conversa muito franca com meu parceiro, de ele não esperar que seja a mesma pessoa de dois anos e meio atrás, que a gente se aventurava, pois essa pessoa não existe mais, por mais que a gente tenha lembranças boas daquela época.* (E31)

Esses achados evidenciam a relevância da sexualidade na vida das participantes, reconhecida como um aspecto que contribui para a saúde e para a qualidade do relacionamento. Observou-se que, no puerpério, a vida sexual tende a ser negligenciada, em decorrência de fatores como a redução do desejo, o medo, a dor, o cansaço e a falta de tempo, relacionados às demandas e às responsabilidades desse período. Ainda assim, foi reconhecida a importância da intimidade do casal, destacando-se o diálogo como elemento fundamental para o enfrentamento das adversidades vivenciadas nessa fase.

### Influência dos Tipos de Parto na Vivência da Sexualidade no Puerpério

A partir das entrevistas, foi possível identificar questões relacionadas à sexualidade das puérperas associadas à via de nascimento e às suas possíveis repercussões. Muitas participantes relataram influência negativa do parto vaginal ou da cesariana relacionada à dor durante a relação sexual, ao medo de retomar a atividade sexual, à redução da libido, à diminuição da lubrificação e às dificuldades na recuperação de lacerações ou episiotomias. Outras intercorrências, como hematomas e hemorroidas, também foram mencionadas como fatores que impactaram a experiência sexual. Além disso, emergiram inseguranças relacionadas à imagem corporal, especialmente em relação aos aspectos estéticos da genitália.

*Tiveram que dar ponto e desenvolvi hemorroida. Isso implicou de forma negativa na minha sexualidade.* (E4)

*Tive tanto episiotomia quanto laceração, então fiquei com muito desconforto e, após um ano, ainda sinto um pouco de desconforto.* (E20)

[...] *eu não sabia como estava lá embaixo, tinha vergonha também do meu corpo, que se juntava com meu medo de machucar* [...] *era vergonha da região ali mesmo, de como estava, eu não sabia como estava*. (E37)

As participantes expressaram insatisfação com a vida sexual no pós-parto, associada a fatores como dor, diminuição da lubrificação, redução da libido e mudanças corporais. Além disso, as que tiveram parto vaginal relataram insegurança em relação à aparência da genitália, enquanto as que se submeteram à cesariana mencionaram desconfortos relacionados à cicatriz, incluindo alteração da sensibilidade e preocupação com possíveis impactos no relacionamento conjugal.

[A cesárea] *me vem que é algo agressivo, me deixou limitada por um tempo. É difícil conviver com algo que demorou para cicatrizar. Aquela região eu não sinto ainda, tive essa perda de sensibilidade na cicatriz. Sinto como se fosse uma ferida que não foi só física, também fiquei pra baixo, chateada, afetou o emocional também.* (E32)

### Orientações Sobre a Sexualidade no Puerpério

Em relação às orientações sobre a sexualidade no pós-parto, observou-se que essa temática ainda não é abordada de forma sistemática nas consultas de acompanhamento ou no contexto da parentalidade. Aproximadamente 43,2% das participantes relataram não ter recebido qualquer orientação profissional sobre o retorno à atividade sexual. Diante dessa lacuna assistencial, muitas pessoas que vivenciaram o puerpério relataram que recorreram à internet, a amigos e a familiares para esclarecer dúvidas. Esses achados sugerem fragilidades na abordagem da sexualidade no cuidado puerperal, evidenciando a necessidade de maior inserção dessa temática nas ações de educação em saúde e no acompanhamento pós-parto.

*Não recebi* [orientação]. *O conhecimento que eu tinha sobre isso se deve ao fato de eu ter estudado sobre isso.* (E9)

*Tive dúvidas, sanei por meio do grupo de apoio do GAPP* [Grupo de Apoio ao Pós-Parto] *e de reuniões virtuais sobre o assunto.* (E18)

Evidenciou-se que os profissionais de saúde tendem a limitar a abordagem ao uso de métodos contraceptivos, com menor exploração de aspectos mais amplos da sexualidade no pós-parto. As participantes destacaram a importância de uma abordagem que normalize as mudanças vivenciadas após o parto e a necessidade de estabelecer um vínculo e uma relação de confiança com o profissional de saúde. Observou-se que apenas 43,2% das entrevistadas receberam algum tipo de orientação, que, na maioria dos casos, se restringiu à recomendação de “resguardo” por 40 dias.

*Na verdade, só me recomendaram tomar o anticoncepcional... foi isso. Me falaram isso no pós-parto; assim que ele nasceu, a enfermeira me orientou.* (E34)

*Só recebi a orientação de esperar os 40 dias, nada além disso. A obstetra me orientou no momento da alta hospitalar. Não fui orientada no pré-natal e em outros momentos, por exemplo.* (E27)

Algumas participantes relataram ter vivenciado dúvidas relacionadas ao retorno à atividade sexual, porém optaram por não buscar esclarecimentos, aguardando o momento em que se sentissem mais confortáveis para retomar essa atividade de forma gradual. Uma participante mencionou não ter seguido o período tradicional de resguardo de 40 dias, optando por retomar a vida sexual quando se sentiu preparada e emocionalmente segura.

*Tive dúvidas. Tive dúvida se teria um tempo adequado para voltar à atividade sexual, se, pela cesárea, meu corpo estaria preparado depois de certo tempo. Essas foram as principais dúvidas. Eu não sanei essas dúvidas, eu esperei meu corpo. Quando eu me senti segura, quando eu não sentia desconforto quando tentávamos, aí acabou acontecendo, mas não busquei informação, nem consulta, fui observando meu corpo e como eu reagia.* (E26)

*Não tive dúvidas, e, inclusive, não segui a orientação de esperar os 40 dias. Pra mim, quando a mulher já se sente bem e está tranquila com isso, pode voltar à vida sexual quando ela sentir vontade.* (E27)

Os achados deste estudo evidenciam que a sexualidade no puerpério ainda é pouco abordada nas consultas da Atenção Primária à Saúde e no contexto da parentalidade, com o predomínio de orientações voltadas aos métodos contraceptivos. Observa-se, ainda, fragilidade nas orientações relacionadas ao período de resguardo e ao retorno à atividade sexual. Nesse cenário, muitas participantes buscam esclarecimentos em fontes informais, como a internet, amigos e familiares, o que pode contribuir para a manutenção de dúvidas e inseguranças. Tais resultados reforçam a necessidade de ampliar a abordagem da sexualidade no cuidado puerperal, incorporando-a de forma sistemática nas ações de educação em saúde e na assistência pós-parto.

## DISCUSSÃO

### Percepções Sobre as Alterações na Sexualidade Durante o Puerpério

A sexualidade no puerpério foi reconhecida pelas participantes como um aspecto que transcende o ato sexual e a reprodução, sendo considerada essencial para a vida das pessoas, independentemente da faixa etária. Entretanto, há uma contradição entre esse entendimento e a prática, pois muitas ainda restringem a sexualidade ao coito penetrativo^(15)^.

No contexto puerperal, destaca-se a relevância da disfunção sexual feminina, que envolve alterações no desejo, na excitação, no orgasmo e na dor sexual^(16)^. As mudanças hormonais, físicas e psicoemocionais desse período podem impactar a vida sexual, sendo a disfunção frequentemente associada a fatores como episiorrafia, lacerações e depressão pós-parto^(17)^. Esses aspectos dialogam com os relatos das entrevistadas, que majoritariamente descreveram a vida sexual de forma negativa. A redução da libido também foi associada à amamentação, atividade frequentemente relatada como cansativa e que pode gerar desconforto quando associada ao estímulo sexual. Considerando que as mamas possuem valor erótico, sua função prioritariamente materna nesse período pode influenciar a vivência da sexualidade. Nesse contexto, torna-se importante que a puérpera consiga diferenciar estímulos maternos e sexuais, além de desconstruir crenças que associam a sexualidade à impureza^(18)^.

Fatores socioculturais, incluindo experiências de socialização, cultura e religião, influenciam a sexualidade ao longo da vida^(19)^. No puerpério, as demandas relacionadas aos filhos podem dificultar os momentos de privacidade e a intimidade do casal^(20)^. Paralelamente, observa-se que algumas pessoas atribuem significados negativos à sexualidade e relatam dificuldades em integrar diferentes dimensões identitárias associadas ao cuidado, ao corpo e à vida sexual. Em contrapartida, também foi percebida pressão social e conjugal para o retorno precoce à atividade sexual. Nesse contexto, evidenciam-se tensões e contradições associadas às construções sociais de gênero, uma vez que, historicamente, a sociedade estabelece normas e padrões que podem reforçar desigualdades e conflitos na vivência da sexualidade^(21)^.

Além disso, os achados que evidenciam a manutenção de relações sexuais para satisfazer o parceiro ou evitar a infidelidade podem ser compreendidos à luz das relações de poder e gênero, nas quais persistem normas sociais que influenciam a vivência da sexualidade no período puerperal. Um estudo recente aponta que a retomada da atividade sexual após o parto é permeada por processos de negociação no âmbito das relações afetivas. Nesses processos, o sexo pode ser socialmente entendido como uma expectativa do relacionamento, com possíveis repercussões sobre a autonomia sexual e a saúde sexual e emocional das pessoas envolvidas^(22)^.

O cônjuge exerce papel relevante no suporte emocional e prático, embora nem sempre de forma efetiva, podendo gerar dificuldades de comunicação e conflitos. A adaptação à parentalidade impõe desafios à dinâmica conjugal e à vida sexual. Dessa forma, destaca-se a importância do apoio familiar e profissional durante o ciclo gravídico-puerperal, com ações educativas sobre as mudanças fisiológicas e emocionais do puerpério e seus impactos na sexualidade^(23)^.

Adicionalmente, é importante considerar que o intervalo de tempo transcorrido entre o puerpério e a coleta dos dados pode ter influenciado as percepções relatadas pelas participantes. Embora a rememoração das experiências permita a elaboração e a ressignificação dos eventos vivenciados, também pode estar sujeita a vieses de memória, uma vez que relatos retrospectivos envolvem processos de reconstrução e interpretação ao longo do tempo, podendo resultar em atenuações ou intensificações das experiências vividas. Nesse sentido, os relatos podem refletir tanto o vivido naquele momento quanto interpretações posteriormente elaboradas, o que deve ser considerado na análise dos achados^(24)^.

### Influência dos Tipos de Parto na Vivência da Sexualidade no Puerpério

Diversos fatores influenciam o tempo de retorno à atividade sexual no puerpério, como comorbidades maternas, medo de nova gestação, medo da dor, grau de lesão perineal e amamentação^(25)^. Entre as entrevistadas, observou-se a percepção de que a via de parto pode impactar a sexualidade, especialmente devido a lacerações ou episiotomias, que podem levar à dispareunia, ao medo, à redução da libido e da lubrificação. Além disso, foram mencionadas inseguranças relacionadas à cicatriz da cesariana e às alterações na genitália.

O parto vaginal pode ocasionar alterações genitais, como trauma perineal e dispareunia, embora apresente benefícios à saúde materna e neonatal, com possíveis intercorrências passíveis de tratamento fisioterapêutico. A cesariana, por sua vez, pode gerar desconforto na cicatriz e impactar a função sexual, embora não haja associação direta entre o tipo de parto e a função sexual^(26)^. Evidencia-se que pessoas submetidas à cesariana ou ao parto vaginal operatório com laceração ou episiotomia podem levar maior tempo para retomar a atividade sexual, sendo a presença de parceiro fixo um fator facilitador^(27)^.

Nesse contexto, destaca-se a relevância do movimento de humanização do parto, que busca reduzir a medicalização e as violências obstétricas, promovendo o protagonismo feminino e o reconhecimento de seus direitos. No âmbito da sexualidade, intervenções como a episiotomia podem resultar em trauma perineal, dispareunia e possíveis disfunções sexuais, enquanto lacerações podem ocorrer fisiologicamente no trabalho de parto. Ambas podem ser prevenidas ou minimizadas com fisioterapia pélvica e técnicas de alongamento perineal, ainda que tais práticas sejam pouco incentivadas, apesar de seu caráter minimamente invasivo^(28)^.

Os relatos também evidenciaram insatisfação com a imagem corporal após o parto, relacionada tanto às alterações decorrentes do parto vaginal quanto à cicatriz da cesariana. As mudanças estéticas corporais, embora esperadas, podem gerar frustração e impactar a autoimagem e a vida sexual das pessoas que vivenciaram o puerpério^(29)^. Assim, observa-se que diferentes vias de parto podem influenciar a experiência sexual no período puerperal, reforçando a importância de orientações e espaços de diálogo sobre o tema, bem como do acesso a informações qualificadas sobre o corpo, a sexualidade e os cuidados nesse contexto.

Nesse sentido, a autoimagem corporal é um importante determinante psicológico da sexualidade, podendo preceder e influenciar o desejo sexual. Evidências recentes indicam que a percepção negativa do próprio corpo no pós-parto está associada à redução da autoestima, à diminuição da satisfação sexual e à maior vulnerabilidade emocional, reforçando a necessidade de abordagens que considerem a subjetividade feminina nesse período^(30)^.

### Orientações Sobre a Sexualidade no Puerpério

O foco das consultas puerperais permanece centrado na concepção, com pouca inclusão do diálogo com os parceiros^(31)^. Considerando os cinco parâmetros de saúde do indivíduo, tornase essencial abordar a sexualidade e seu impacto na qualidade de vida da puérpera e do casal^(32)^. As entrevistas evidenciam a carência de informações profissionais que validem como normais as experiências vivenciadas nesse período, reforçando a necessidade de orientação qualificada.

Adicionalmente, é importante considerar que o perfil das participantes, majoritariamente mulheres brancas e com maior nível de escolaridade, pode influenciar o acesso à informação e à assistência à saúde. A literatura aponta que marcadores sociais, como raça, classe e escolaridade, compreendidos na perspectiva da interseccionalidade, impactam o acesso aos serviços de saúde e a qualidade do cuidado recebido, sendo que mulheres em contextos de maior vulnerabilidade podem enfrentar barreiras ainda mais significativas no acesso a orientações qualificadas^(33)^.

Diante da influência da internet na comunicação e na educação em saúde, esse meio é frequentemente utilizado para o esclarecimento de dúvidas. Entretanto, as informações obtidas podem gerar insegurança e impactar negativamente a vivência da sexualidade. Questões relacionadas à atividade sexual ainda suscitam dúvidas entre puérperas, associadas ao constrangimento e ao receio em abordar o tema com profissionais de saúde^(34)^.

Reconhece-se a importância de fornecer informações ao casal e capacitar profissionais para tratar da sexualidade no puerpério. No Brasil, o Ministério da Saúde dispõe dos Cadernos de Atenção Básica em Saúde Sexual e Reprodutiva, que orientam práticas qualificadas nesse campo^(35)^. O método *Permission, Limited Information, Specific Suggestions, Intensive Therapy* é destacado como um modelo de consulta aplicável pelo enfermeiro no manejo das disfunções sexuais. As recomendações enfatizam a consideração do contexto sociocultural, a oferta de suporte emocional e psicológico e a adoção de abordagens não farmacológicas, com educação sexual de qualidade. Ademais, o aconselhamento deve iniciar no pré-natal, identificando disfunções e orientando sobre mudanças fisiológicas para preparar o casal para o ciclo gravídico-puerperal^(36)^.

Percebe-se a negligência dos profissionais de saúde, inclusive dos enfermeiros, ao abordarem a sexualidade, o que se reflete na escassez de estudos sobre intervenções terapêuticas. A discussão do tema pode favorecer uma comunicação mais aberta entre gestantes, puérperas e profissionais de saúde, além de estimular pesquisas futuras. Desse modo, é essencial capacitar os enfermeiros, especialmente, pois a sexualidade é um pilar do cuidado em saúde. A equipe deve atuar de maneira eficaz desde o pré-natal, considerando as particularidades de cada pessoa, para superar dificuldades relacionadas à sexualidade^(37)^.

Embora o número de participantes seja adequado à proposta do estudo, ele não permite generalizações, considerando a magnitude da população investigada. Ressalta-se, contudo, que a pesquisa qualitativa busca compreender o fenômeno a partir das narrativas e singularidades das participantes. Assim, sugere-se a realização de novos estudos com estratégias de busca ativa e ampliação da divulgação, associadas ao método bola de neve, para maior representatividade das puérperas.

Entre as limitações do estudo, destaca-se, inicialmente, o intervalo temporal entre a experiência do puerpério, a coleta dos dados (2021-2022) e o período de publicação, o que pode ter influenciado os relatos a partir de processos de rememoração, elaboração e ressignificação das experiências vividas. Ainda que a memória permita reflexões mais amadurecidas sobre o vivido, relatos retrospectivos estão sujeitos a vieses, podendo atenuar ou intensificar determinadas percepções.

Outra limitação refere-se ao perfil das participantes, composto majoritariamente por mulheres brancas, com maior nível de escolaridade e residentes em contexto urbano. Esses marcadores sociais de raça, classe e gênero podem influenciar o acesso à informação, aos serviços de saúde e às possibilidades de cuidado, limitando a extrapolação dos resultados para mulheres em contextos de maior vulnerabilidade social, racial e econômica.

Adicionalmente, embora o número de 37 participantes tenha permitido identificar recorrências temáticas e alcançar saturação dos dados, trata-se de um contingente elevado para estudos qualitativos que demandam maior densidade analítica em profundidade individual, o que pode ter limitado um aprofundamento mais minucioso de cada narrativa. Essa limitação é potencializada pelo uso combinado de entrevistas dialógicas e formulários autoaplicáveis, uma vez que o segundo formato restringe a possibilidade de exploração aprofundada dos sentidos emergentes quando comparado à entrevista síncrona.

Como contribuição para a enfermagem, espera-se que o estudo incentive o aprimoramento profissional e amplie a abordagem da sexualidade feminina, desde o pré-natal até o puerpério, estimulando o diálogo sobre a temática nos diferentes espaços de atuação do enfermeiro. Além disso, os resultados podem subsidiar novas pesquisas e contribuir para a qualificação das ações de educação permanente em saúde.

## CONCLUSÃO

A pesquisa compreendeu a vivência da sexualidade no puerpério, evidenciando que, para muitas pessoas, exercer a sexualidade de forma satisfatória nesse período constitui um desafio. Embora reconheçam sua importância para a saúde, a autoestima e a manutenção de relacionamentos saudáveis, as demandas da parentalidade frequentemente reduzem o tempo destinado à intimidade, tornando o diálogo entre o casal fundamental para a preservação do vínculo.

Fatores biológicos, como dor durante as relações, redução da libido, efeitos da amamentação e insatisfação corporal, foram apontados como limitantes para o retorno ao padrão sexual anterior à gestação. Além disso, tanto o parto vaginal quanto a cesariana foram associados a desconfortos que podem impactar a sexualidade. Evidenciou-se, ainda, fragilidade na abordagem da sexualidade pelos profissionais de saúde durante o ciclo gravídico-puerperal, com a predominância de orientações restritas aos métodos contraceptivos e ao período de resguardo.

O estudo reforça a necessidade de ampliar a abordagem da sexualidade no cuidado em saúde, especialmente na enfermagem, desde a formação acadêmica até a prática assistencial, promovendo educação em saúde e suporte qualificado às pessoas que vivenciam o puerpério.

## Data Availability

Todo o conjunto de dados que dá suporte aos resultados deste estudo foi disponibilizado em: https://repositorio.ufsc.br/handle/123456789/248224.

# Translation: La experiencia de la sexualidad en el posparto: estudio cualitativo en una ciudad del sur de Brasil

**DOI:** 10.1590/1980-220X-REEUSP-2026-0033es

**Authors:** Viviane Vanderlinde da Silva, Matheus da Cunha Paris, Manuela Beatriz Velho, Luiza Todeschini Vieira, Beatriz de Souza Rodrigues, Laís Antunes Wilhelm

**Affiliations:** 1 Universidade Federal de Santa Catarina, Departamento de Enfermagem, Florianópolis, SC, Brasil.

**Keywords:** Sexualidad, Periodo Posparto, Enfermería, Investigación Cualitativa

## Abstract

**Objetivo:**

Comprender la experiencia de la sexualidad en el periodo posparto.

**Método:**

Estudio cualitativo, realizado mediante dos estrategias de recolección de datos distintas y complementarias — entrevistas semiestructuradas y un cuestionario autoadministrado con preguntas abiertas —, ambos elaborados a partir del mismo guion. Participaron 37 personas que vivieron el periodo posparto en el estado de Santa Catarina entre el segundo semestre de 2021 y finales de 2022. Se adoptó un análisis temático de los datos obtenidos.

**Resultados:**

Se evidenció que las personas entrevistadas consideran importante la sexualidad, pero durante el posparto experimentaron dificultades para mantener relaciones sexuales debido a fatiga, falta de libido y dolor durante el coito, entre otros factores relacionados con este periodo.

**Conclusión:**

Se concluye que el tema de la sexualidad no se aborda con regularidad en las consultas posparto, limitándose a tratar los métodos anticonceptivos y el posparto en sí.

## INTRODUCCIÓN

La parentalidad, entendida como un proceso que incluye el puerperio, constituye una etapa singular en la vida de las personas que gestan y viven este período, abarcando dimensiones individuales, familiares y sociales^(1)^. El puerperio, también denominado posparto, comienza con la salida del feto y se extiende hasta el término de los procesos involutivos de la gestación, clasificándose en tres fases: inmediata (del 1.º al 10.º día), tardía (del 11.º al 40.º día) y remota (a partir del día 41, sin un final definido)^(2)^.

En este período, la persona que atraviesa el puerperio experimenta diversas transformaciones físicas, emocionales, conductuales y sexuales. La sexualidad va más allá del acto sexual y la reproducción, abarcando la identidad de género, la orientación sexual, el placer, las creencias, las conductas y las relaciones interpersonales. Está influenciada por factores biológicos, psicológicos, sociales, económicos, culturales, históricos y religiosos, lo que la convierte en un aspecto esencial de la vida humana^(3)^.

Cabe destacar la importancia de ampliar el conocimiento sobre la sexualidad en el posparto, etapa en la que las personas pueden presentar una mayor vulnerabilidad debido a posibles complicaciones de salud y a una menor atención en comparación con otras etapas del ciclo gravídico-puerperal. La evidencia indica que, a pesar de la relevancia del tema, la sexualidad en el puerperio sigue siendo poco explorada en los servicios de salud, y con frecuencia se descuida y se aborda de manera superficial^(4)^.

Además, se observa que el tema suele quedar invisibilizado incluso en la etapa prenatal, período en el que predominan las orientaciones centradas en los aspectos biológicos del embarazo, el parto y los cuidados del recién nacido, con un enfoque limitado de las dimensiones sexuales y relacionales de las personas que están embarazadas y viven el puerperio^(5)^. Esta brecha refleja, en parte, la falta de preparación de los profesionales de la salud para abordar la sexualidad de manera integral, ya sea por la insuficiencia de capacitación específica, por la presencia de tabúes socioculturales o por dificultades en la comunicación con las usuarias^(6)^.

En este contexto, el personal de enfermería desempeña un papel fundamental al promover el diálogo sobre la sexualidad, contribuyendo a aclarar dudas y a que la experiencia de este período sea más acogedora^(7)^. Sin embargo, el estudio señala la necesidad de capacitación profesional y de incorporar prácticas educativas sistematizadas que consideren la sexualidad como un componente esencial de la atención integral de la salud de las personas que están embarazadas y atraviesan el puerperio^(6)^.

Cabe destacar la Política Nacional de Derechos Sexuales y Reproductivos, establecida por el Ministerio de Salud en 2005^(8)^, que fomenta acciones educativas en materia de salud sexual y reproductiva, y los Objetivos de Desarrollo Sostenible de la Agenda 2030, especialmente en lo que se refiere a garantizar el acceso universal a la salud sexual y reproductiva y a los derechos reproductivos^(9)^. En este sentido, ampliar el enfoque de la sexualidad durante el puerperio contribuye a mejorar la calidad de la atención de salud y a fortalecer las políticas públicas orientadas a la equidad, la atención integral y la promoción de los derechos de las personas que están embarazadas y atraviesan el puerperio^(10)^.

A pesar de ello, la sexualidad, especialmente en el posparto, sigue siendo poco abordada en los servicios de salud, que con frecuencia adoptan un modelo biologicista centrado en la reproducción y la anticoncepción. Esto pone de manifiesto la necesidad de una mayor capacitación de los profesionales y de fortalecer el vínculo con las usuarias^(10)^.

Ante este contexto, el objetivo de este estudio fue comprender la experiencia de la sexualidad en el posparto.

## MÉTODO

### Diseño del Estudio

Se trata de una investigación de campo con enfoque cualitativo, que forma parte del macroproyecto titulado “Sexualidad femenina en el posparto: experiencias de las mujeres”. Se considera necesario el enfoque cualitativo para cumplir con los objetivos del estudio, ya que se trata de valores y creencias de los actores sociales^(11)^. Cabe destacar, además, que la investigación se guió por los *COnsolidated criteria for REporting Qualitative research*, que proporcionan directrices para la planificación, la ejecución y la elaboración de informes de investigaciones cualitativas^(12)^.

### Lugar

El estudio se llevó a cabo en los municipios del área metropolitana de Florianópolis, en Santa Catarina.

### Población y Criterios de Selección

La población del estudio estuvo compuesta por personas que vivieron el puerperio a partir de 2017, mayores de 18 años y con vida sexual activa, con el fin de garantizar una mayor fiabilidad de los recuerdos. Se excluyeron del estudio las personas que tuvieron un embarazo de alto riesgo o que vivieron un embarazo en la adolescencia, considerando las posibles particularidades de estas experiencias.

### Recolección de Datos y Definición de la Muestra

La recolección de datos se llevó a cabo mediante dos estrategias distintas y complementarias —entrevistas semiestructuradas y un cuestionario autoadministrado con preguntas abiertas—, ambas elaboradas a partir del mismo guion de preguntas, con el fin de garantizar la coherencia temática entre los diferentes modos de producción de los datos.

Las entrevistas semiestructuradas se realizaron de manera sincrónica y a distancia a través de la plataforma *Google Meet* ^®^, caracterizándose como un proceso dialógico entre la investigadora y la participante. Este formato permitió profundizar más en las narrativas, explorar significados y aclarar los sentidos que surgían a partir de la interacción, de acuerdo con los supuestos de la investigación cualitativa. Las entrevistas, que tuvieron una duración promedio de 40 minutos, se grabaron con la autorización de las participantes y, posteriormente, se transcribieron en su totalidad para su análisis.

Paralelamente, se utilizó un formulario autoadministrado con preguntas abiertas, disponible a través de la plataforma *Google Forms* ^®^. A diferencia de la entrevista, el formulario no supone una interacción dialógica, sino que constituye un instrumento escrito de autorrelato. La adopción de esta estrategia tuvo como objetivo ampliar el alcance de la investigación y favorecer la participación de personas que vivieron el puerperio y que manifestaban temor o incomodidad al abordar abiertamente cuestiones relacionadas con la sexualidad en un contexto interactivo, sobre todo considerando el carácter íntimo del tema investigado. El tiempo promedio para completar el formulario fue de aproximadamente 20 minutos.

La difusión del estudio y el reclutamiento de las participantes se realizaron a través de aplicaciones de mensajería y redes sociales, como *WhatsApp* ^®^ e *Instagram* ^®^. De este modo, se utilizó la técnica de muestreo en bola de nieve, indicada para investigaciones que abordan temas delicados y poblaciones de difícil acceso, así como para contextos de amplia dispersión geográfica^(13)^.

Participaron en el estudio 37 personas que vivieron la situación, de las cuales 14 a través de entrevistas sincrónicas y 23 mediante un formulario de autoevaluación. El número de participantes se definió a partir del criterio de saturación de los datos, considerando la recurrencia de significados en las narrativas. La recolección de datos se llevó a cabo entre el segundo semestre de 2021 y finales de 2022.

Al acceder al cuestionario o antes de la entrevista, las participantes tuvieron acceso al Término de Consentimiento Libre e Informado (TCLE), pudiendo aceptar o rechazar su participación. Tras la aceptación, se recopiló información sociodemográfica y obstétrica, como edad, municipio de residencia, estado civil, nivel educativo, número de embarazos e hijos, color/raza autodeclarada, religión y datos relacionados con el último embarazo. Tras la etapa sociodemográfica, se presentó la pregunta inicial “¿Qué significa la sexualidad para ti?”, seguida de preguntas complementarias relacionadas con la experiencia de la sexualidad antes, durante y después del embarazo. Para este estudio, se utilizó el enfoque referido al período puerperal. El instrumento de recolección se probó previamente mediante una entrevista piloto, sin que fuera necesario realizar ajustes.

### Análisis y Procesamiento de Datos

El análisis de los datos se llevó a cabo de acuerdo con la propuesta operativa de Minayo, que contempla dos aspectos: el histórico-social y el interpretativo. Este enfoque se organiza en dos niveles operativos: el histórico-social, que tomó en cuenta las características del grupo investigado, incluyendo aspectos socioeconómicos, y el interpretativo, que buscó identificar, en los relatos, significados, lógicas e interpretaciones relacionadas con la sexualidad en el puerperio. Para llevar a cabo el segundo nivel, fue necesario ordenar y clasificar los datos y las respuestas obtenidas en las entrevistas^(14)^.

La primera etapa consistió en la transcripción y relectura del material obtenido. En la segunda etapa, los datos se clasificaron mediante una lectura horizontal, destinada a identificar las impresiones iniciales y las ideas centrales, seguida de una lectura transversal, que permitió profundizar y comprender con mayor detalle el material empírico. Posteriormente, se llevó a cabo el análisis final, articulado con la literatura científica, lo que permitió responder a la pregunta guía del estudio y alcanzar el objetivo propuesto^(14)^.

A partir de los resultados, surgieron tres categorías distintas: “Percepciones sobre los cambios en la sexualidad durante el puerperio”; “Influencia de los tipos de parto en la experiencia de la sexualidad en el puerperio”; y “Orientaciones sobre la sexualidad en el puerperio”. Para garantizar el anonimato de las participantes, se utilizó la letra “E”, seguida del número correspondiente a su participación en la investigación.

### Aspectos Éticos

El macroproyecto fue aprobado por el Comité de Ética en Investigación de la Universidad Federal de Santa Catarina el 29 de marzo de 2021 (Dictamen n.º 4.619.478 y Certificado de Presentación para Evaluación Ética n.º 42605020.0.0000. 0121), al que está vinculado el presente estudio. El TCLE se puso a disposición antes de la Recolección de datos y se envió a las participantes por vía electrónica. Se respetaron todos los principios éticos para la investigación con seres humanos, y todas las participantes firmaron el TCLE.

## RESULTADOS

El perfil de las participantes muestra que la mayoría tenía entre 21 y 40 años, con un predominio de los grupos de edad de 21 a 25 años (24,3 %) y de 31 a 35 años (24,3 %). En cuanto al lugar de residencia, el 62,1 % residía en Florianópolis. En cuanto a la identidad de género, todas se identificaron como mujeres cisgénero (100 %). Predominaron las participantes casadas (56,7 %), con estudios superiores completos (32,4 %) o incompletos (29,7 %). En cuanto a la raza o color autodeclarado, el 62,2 % eran blancas, el 21,6 %, mestizas, el 13,5 %, negras y el 2,7 %, indígenas. En cuanto a la religión, se observó una proporción similar entre católicas y sin religión (29,7 %).

Al momento de la entrevista, la mayoría (94,6 %) se encontraba en el posparto remoto. En cuanto al número de embarazos, el 67,6 % tuvo un embarazo y el 32,4 % tuvo dos o más. En cuanto al tipo de parto, el 62,2 % tuvo un parto vaginal y el 35,1 %, una cesárea. Entre los partos vaginales, el 82,6 % presentó laceración espontánea, el 13,1 %, episiotomía, y el 4,3 % no reportó complicaciones.

Se observó que el 76,5 % de las participantes indicaron no haber recuperado el patrón sexual anterior al embarazo, y el 59,5 % consideraron que su vida sexual actual era peor en comparación con el período previo al embarazo.

Del análisis de los datos surgieron tres categorías: “Percepciones sobre los cambios en la sexualidad durante el puerperio”; “Influencia de los tipos de parto en la experiencia de la sexualidad durante el puerperio”; y “Orientaciones sobre la sexualidad durante el puerperio”.

### Percepciones Sobre los Cambios en la Sexualidad Durante el Puerperio

Las participantes describieron la sexualidad como un elemento fundamental para la calidad de vida, asociándola a términos como “conexión” (con una misma y con la otra persona), “alianza”, “placer”, “emoción”, “atracción física y afectiva”, “deseo”, “cariño”, “sexo/acto sexual” y “salud”. En cuanto a su importancia, la sexualidad se consideró esencial, con influencia sobre el estado de ánimo, la salud y la energía creativa, y se percibió incluso como tan relevante para la relación como el respeto y la fidelidad, configurándose como un pilar para las relaciones saludables.

*Afecta el estado de ánimo y la disposición para otras actividades.* (E6)

*Para mí, la sexualidad es importante para el equilibrio de nuestro cuerpo, mente y salud, ya que, cuando la practicamos, nos sentimos mejor y más saludables.* (E13)

*Es importante para la autoestima; relaja y une a la pareja.* (E18)

Algunas participantes relataron que la sexualidad ya había sido más relevante en sus vidas, pero mencionaron una disminución del interés y de la necesidad, que atribuyeron a las múltiples exigencias relacionadas con la parentalidad, el trabajo, las tareas domésticas y los estudios. El cansancio se mencionó como factor predominante en esta etapa, lo que contribuyó a que la intimidad de la pareja pasara a un segundo plano. Además, algunas participantes mencionaron que comparten la cama con el bebé, lo que sugiere que esta práctica influye en su vida sexual.

*No tengo ganas... el cansancio me domina. Todavía estoy amamantando. Y tengo miedo de que, durante el acto, el bebé se despierte y se acabe todo el ambiente.* (E6)

[La vida sexual está] *lejos de ser una prioridad, aunque me siento bien cuando la practico.* (E8)

*Hoy quiero cuidar a mi hija, estabilizarme económicamente y estudiar, así que estoy tan enfocada en otras cosas que eso* [la vida sexual] *termina quedando en segundo plano* [...] *después de que ella nació, ya no retomé eso, sobre todo porque duerme conmigo en la cama. No me siento bien, y soy yo quien la cuida todo el tiempo y me gusta hacerlo sola. Así que, sin duda, la maternidad ha influido mucho en mi sexualidad.* (E33)

Una participante destacó que, después del parto, comenzó a percibir la sexualidad como una expresión de cariño, mientras que antes la entendía predominantemente como un acto físico.

[...] *después de tener a mis hijas, la sexualidad para mí ya no es solo el acto sexual, se ha convertido mucho más en un intercambio de caricias, el afecto que tengo para dar, y solo así tener y desear el acto sexual. Antes no necesitaba cariño ni afecto, era simplemente el acto, y esa es la mayor diferencia que percibo ahora después del parto.* (E29)

Otra participante comentó que creía que la sexualidad había dejado de ser tan importante en su vida, lo cual asoció con la duración de la relación.

*Ya hubo más; ahora estoy en una etapa en la que ya no es tan importante* [...] *no sé si es porque tuve a mi hijo o por el tiempo que llevamos juntos y conviviendo. Nunca había estado tanto tiempo con una persona... creo que también puede ser eso.* (E24)

En cuanto a los cambios en la sexualidad, algunas mencionaron dolor y molestias durante la penetración. Sin embargo, a través del autoconocimiento y el diálogo con su pareja, comprendieron la necesidad de un mayor estímulo para alcanzar el placer sexual durante el puerperio. Cabe destacar que esta experiencia se relató incluso años después del parto.

*Pero fue difícil. En esos meses, pensé que el dolor se debía a que estaba un poco apretada, pero cuando me relajé, me di cuenta de que era porque yo estaba tensa. Cuando me relajaba, ya no me dolía el músculo, así que le decía a mi esposo que me estimulara bastante para que pudiera relajarme.* (E29)

*Tenía ganas de volver pronto* [a la actividad sexual], *hasta que descubrí que me dolía mucho. No veía la hora de que llegaran los 40 días* [de reposo]. *Estuve esperando a que se me pasara el dolor. Pensé que se me pasaría pronto, pero nunca se me pasó en estos tres años.* (E24)

Las entrevistadas también mencionaron la percepción de “presión” por parte de sus parejas y de la sociedad para volver pronto a la actividad sexual. Algunas mencionaron tener relaciones sexuales con el objetivo de complacer a su pareja, incluso cuando sentían molestias. Además, una participante contó que había tenido sexo con penetración por miedo a la atrofia muscular, lo que pone de manifiesto un desconocimiento sobre el funcionamiento del cuerpo femenino.

*No tenía ganas en absoluto de tener relaciones sexuales y mi esposo empezó a sentir la necesidad y me lo pedía, y yo hasta lo hacía para satisfacerlo, pero era muy horrible, totalmente incómodo* [...]*no siento ganas en absoluto, pero él es el papá de mi hija.* (E4)

*Cuando tuve mi primera relación sexual* [después del parto]*, fue más que nada por la presión* [...] *si fuera hoy, habría esperado más a que llegara mi momento. Cuando estaba embarazada, mi papá me dijo lo siguiente cuando le comenté que quería un parto natural: “Le vas a arruinar todo el juguetito al hombre”. Mi papá tiene 56 años y es machista. Y después de que dijo eso, me quedé muy pensativa, porque estaba al inicio del embarazo, y ya no quise que mi pareja estuviera conmigo en el parto* [...] *nos aferramos mucho a lo que dice la sociedad sobre volver pronto a la actividad sexual, porque si no, el hombre no lo va a aguantar y va a buscar fuera de casa.* (E31)

También se mencionaron otras cuestiones, como la dificultad de separar la vida sexual de las exigencias de la parentalidad, la influencia de la lactancia materna y la percepción de un menor interés por parte de las parejas en mantener la vida sexual tras asumir la paternidad.

*Está siendo una experiencia horrible, parece que la maternidad ha destruido la libido de mi pareja.* [...] *parece que mi pareja ya no siente deseo por mí. Las pocas veces que lo intentamos, después de insistirle un sinfín de veces, el bebé se despierta* [...] [me siento] *muy frustrada.* (E11)

[...] *a veces, vemos el acto sexual como algo sucio, aunque sepamos que es algo natural y que todo el mundo lo hace, pero eso todavía tengo que desmontarlo en mi mente. Tengo esa dualidad. Sé que el acto sexual es algo normal y que todo el mundo lo hace, pero mi lado maternal me hace pensar incluso en cosas como, por ejemplo, mis senos, ya que ella se alimenta de ellos. Esta cuestión de separar a la madre de la mujer es muy difícil.* (E33)

Para describir la vida sexual después del parto, las participantes utilizaron términos como “escasas”, “pésima”, “poco activa”, “sin libido”, “inexistente” y “dolorosa”. Sin embargo, una minoría la describió como similar al período anterior, excelente o activa.

Al comparar la vida sexual durante el embarazo y en el posparto, la mayoría de las participantes (59,5 %) la calificó como peor. Entre los motivos citados, destacan la falta de disponibilidad, la lactancia materna, la disminución del deseo y la presencia de dolor o molestias durante las relaciones sexuales.

Por otro lado, siete participantes informaron una mejora en la vida sexual después del parto, asociando esta percepción a una mayor autonomía, al empoderamiento, al autoconocimiento corporal y al aumento del placer. También se mencionaron factores como dejar de compartir la habitación con el bebé, el fin de la lactancia materna, la práctica de la masturbación y el fortalecimiento del respeto y de la relación conyugal.

*Creo que ha cambiado bastante, incluso en cuanto a la frecuencia, pero ahora me gusta más y disfruto más, en comparación con antes.* (E25)

[...] *noté una mejora en el aspecto sexual después de que la bebé se fuera de nuestra habitación, cuando empezó a dormir en la suya; parece que volvimos a tener una vida de pareja* [...] *una diferencia que veo es incluso en relación con mi pareja, en que él nos ve como mujer de nuevo, no solo como madre.* (E27)

*Cuando tengo ganas, recurro a la masturbación, por ejemplo. A veces, en esos momentos, me encierro en la regadera* [...] *pensando en mí, en sentir placer, me siento bien. Me siento más mujer, más preparada, más involucrada. Conozco mejor mi cuerpo por haber tenido un parto normal, así que todo se desarrolla más fácil, de manera más natural.* (E30)

En cuanto al regreso al patrón de vida sexual anterior al embarazo, 26 participantes (76,5 %) informaron no haber retomado ese patrón. Entre los motivos señalados, destacan la disminución del deseo, la sobrecarga de actividades, la mayor dedicación al cuidado del hijo, la disminución del romanticismo y los cambios corporales. Una participante mencionó que no buscaba ese regreso, ya que considera que las circunstancias actuales son diferentes a las del período anterior al embarazo.

*Ha cambiado mucho. Hasta hoy, cuatro años después, sigue siendo difícil; parece que he perdido las ganas de tener relaciones sexuales, de llevar una vida sexual activa.* (E7)

[...] *otra cosa importante es la prioridad de la pareja, que ha cambiado. Vivimos para los hijos y terminamos olvidándonos mucho de la relación como pareja. Creo que vale la pena señalar esto. Falta romanticismo, falta cariño, y creo que esto debería trabajarse con la pareja, que tiene hijos y se olvida de “ser pareja” y solo piensa en ser papá y mamá.* (E28)

*No ha sucedido, y tampoco busco que suceda. Ya entendí que mi cuerpo es otro, que esta etapa de mi vida es diferente, y lo respeto mucho. Tengo una conversación muy franca con mi pareja, para que no espere que sea la misma persona de hace dos años y medio, cuando nos aventurábamos, porque esa persona ya no existe, por más que tengamos buenos recuerdos de esa época.* (E31)

Estos hallazgos evidencian la importancia de la sexualidad en la vida de las participantes, reconocida como un aspecto que contribuye a la salud y a la calidad de la relación. Se observó que, durante el posparto, la vida sexual tiende a descuidarse, como consecuencia de factores como la disminución del deseo, el miedo, el dolor, el cansancio y la falta de tiempo, relacionados con las exigencias y responsabilidades de este período. Aun así, se reconoció la importancia de la intimidad de la pareja, destacándose el diálogo como elemento fundamental para enfrentar las adversidades vividas en esta etapa.

### Influencia de los Tipos de Parto en la Experiencia de la Sexualidad Durante el Puerperio

A partir de las entrevistas, fue posible identificar cuestiones relacionadas con la sexualidad de las puérperas asociadas a la vía de parto y sus posibles repercusiones. Muchas participantes informaron sobre la influencia negativa del parto vaginal o la cesárea en relación con el dolor durante las relaciones sexuales, el miedo a retomar la actividad sexual, la disminución de la libido, la reducción de la lubricación y las dificultades para recuperarse de desgarros o episiotomías. También se mencionaron otras complicaciones, como hematomas y hemorroides, como factores que afectaron la experiencia sexual. Además, surgieron inseguridades relacionadas con la imagen corporal, especialmente en lo que respecta a los aspectos estéticos de los genitales.

*Me tuvieron que dar puntos y me salió una hemorroide. Eso afectó negativamente mi sexualidad.* (E4)

*Tuve tanto una episiotomía como una laceración, así que sentí mucho malestar y, después de un año, todavía siento un poco de malestar.* (E20)

[...] *no sabía cómo estaba ahí abajo, también me daba vergüenza mi cuerpo, lo cual se sumaba a mi miedo a lastimarme* [...] *era vergüenza de esa zona en particular, de cómo estaba, no sabía cómo estaba*. (E37)

Las participantes expresaron insatisfacción con su vida sexual después del parto, asociada a factores como el dolor, la disminución de la lubricación, la reducción de la libido y los cambios corporales. Además, las que tuvieron un parto vaginal reportaron inseguridad respecto al aspecto de sus genitales, mientras que las que se sometieron a una cesárea mencionaron molestias relacionadas con la cicatriz, incluyendo alteraciones en la sensibilidad y preocupación por posibles repercusiones en la relación de pareja.

[La cesárea] *me parece algo agresivo, me dejó limitada por un tiempo. Es difícil lidiar con algo que tardó en sanar. Todavía no siento esa zona, tuve esa pérdida de sensibilidad en la cicatriz. Siento como si fuera una herida que no fue solo física; también me sentí deprimida, molesta; me afectó emocionalmente también.* (E32)

### Orientación Sobre la Sexualidad en el Puerperio

En cuanto a la orientación sobre la sexualidad después del parto, se observó que este tema aún no se aborda de manera sistemática en las consultas de seguimiento ni en el contexto de la parentalidad. Aproximadamente el 43,2 % de las participantes informaron no haber recibido ninguna orientación profesional sobre el regreso a la actividad sexual. Ante esta brecha en la atención, muchas personas que vivieron el puerperio informaron que recurrieron a Internet, a amigos y a familiares para aclarar sus dudas. Estos hallazgos sugieren debilidades en el enfoque de la sexualidad en la atención posparto, lo que pone de manifiesto la necesidad de una mayor integración de este tema en las acciones de educación en salud y en el seguimiento posparto.

*No recibí* [orientación]. *Lo que sabía sobre esto se debe a que lo estudié.* (E9)

*Tuve dudas, las resolví a través del grupo de apoyo del GAPP* [Grupo de Apoyo al Posparto] *y de reuniones virtuales sobre el tema.* (E18)

Se puso de manifiesto que los profesionales de la salud tienden a limitar el enfoque al uso de métodos anticonceptivos, sin profundizar tanto en aspectos más amplios de la sexualidad en el posparto. Las participantes destacaron la importancia de un enfoque que normalice los cambios que se experimentan después del parto y la necesidad de establecer un vínculo y una relación de confianza con el profesional de la salud. Se observó que solo el 43,2 % de las entrevistadas recibió algún tipo de orientación, la cual, en la mayoría de los casos, se limitó a la recomendación de “cuidado” durante 40 días.

*En realidad, solo me recomendaron tomar anticonceptivos... eso fue todo. Me lo dijeron después del parto; tan pronto como nació, la enfermera me lo explicó.* (E34)

*Solo me aconsejaron esperar los 40 días, nada más. La obstetra me lo explicó al momento del alta hospitalaria. No me dieron orientación durante el prenatal ni en otros momentos, por ejemplo.* (E27)

Algunas participantes relataron haber tenido dudas relacionadas con la reanudación de la actividad sexual, pero optaron por no buscar aclaraciones, esperando el momento en que se sintieran más cómodas para retomar esta actividad de manera gradual. Una participante mencionó que no había seguido el período tradicional de reposo de 40 días, y optó por retomar la vida sexual cuando se sintió preparada y emocionalmente segura.

*Tuve dudas. Tenía dudas sobre si tendría el tiempo adecuado para volver a la actividad sexual, si, debido a la cesárea, mi cuerpo estaría preparado después de cierto tiempo. Esas fueron las principales dudas. No resolví esas dudas, esperé a que mi cuerpo estuviera listo. Cuando me sentí segura, cuando ya no sentía molestias al intentarlo, entonces sucedió, pero no busqué información ni consulté a nadie; simplemente fui observando mi cuerpo y cómo reaccionaba.* (E26)

*No tuve dudas y, de hecho, no seguí la recomendación de esperar los 40 días. Para mí, cuando la mujer ya se siente bien y está tranquila con eso, puede retomar su vida sexual cuando tenga ganas.* (E27)

Los hallazgos de este estudio evidencian que la sexualidad en el puerperio aún se aborda poco en las consultas de atención primaria de salud y en el contexto de la parentalidad, con un predominio de orientaciones enfocadas en los métodos anticonceptivos. Además, se observa una falta de claridad en las orientaciones relacionadas con el período de reposo y el regreso a la actividad sexual. En este contexto, muchas participantes buscan aclaraciones en fuentes informales, como Internet, amigos y familiares, lo que puede contribuir a que persistan las dudas y las inseguridades. Estos resultados refuerzan la necesidad de ampliar el enfoque de la sexualidad en la atención posparto, incorporándola de manera sistemática en las acciones de educación en salud y en la atención posparto.

## DISCUSIÓN

### Percepciones Sobre los Cambios en la Sexualidad Durante el Puerperio

Las participantes reconocieron que la sexualidad en el puerperio es un aspecto que trasciende el acto sexual y la reproducción, y la consideraron esencial para la vida de las personas, independientemente de la edad. Sin embargo, existe una contradicción entre esta comprensión y la práctica, ya que muchas aún limitan la sexualidad al coito penetrativo^(15)^.

En el contexto posparto, se destaca la relevancia de la disfunción sexual femenina, que implica cambios en el deseo, la excitación, el orgasmo y el dolor sexual^(16)^. Los cambios hormonales, físicos y psicoemocionales de este período pueden afectar la vida sexual, y la disfunción se asocia con frecuencia a factores como la episiotomía, las laceraciones y la depresión posparto^(17)^. Estos aspectos concuerdan con los relatos de las entrevistadas, quienes en su mayoría describieron la vida sexual de manera negativa. La disminución de la libido también se asoció con la lactancia materna, actividad que con frecuencia se describe como agotadora y que puede generar incomodidad cuando se asocia con la estimulación sexual. Considerando que los senos tienen un valor erótico, su función principalmente materna en este período puede influir en la experiencia de la sexualidad. En este contexto, es importante que la mujer en el posparto logre diferenciar los estímulos maternos de los sexuales, además de desmontar las creencias que asocian la sexualidad con la impureza^(18)^.

Los factores socioculturales, incluyendo las experiencias de socialización, la cultura y la religión, influyen en la sexualidad a lo largo de la vida^(19)^. En el puerperio, las exigencias relacionadas con los hijos pueden dificultar los momentos de privacidad y la intimidad de la pareja^(20)^. Paralelamente, se observa que algunas personas atribuyen significados negativos a la sexualidad y reportan dificultades para integrar diferentes dimensiones identitarias asociadas al cuidado, al cuerpo y a la vida sexual. Por otro lado, también se percibió una presión social y conyugal para el regreso precoz a la actividad sexual. En este contexto, se evidencian tensiones y contradicciones asociadas a las construcciones sociales de género, ya que, históricamente, la sociedad establece normas y estándares que pueden reforzar las desigualdades y los conflictos en la vivencia de la sexualidad^(21)^.

Además, los hallazgos que evidencian la continuación de las relaciones sexuales para satisfacer a la pareja o evitar la infidelidad pueden entenderse a la luz de las relaciones de poder y género, en las que persisten normas sociales que influyen en la vivencia de la sexualidad durante el período posparto. Un estudio reciente señala que la reanudación de la actividad sexual después del parto está marcada por procesos de negociación en el ámbito de las relaciones afectivas. En estos procesos, el sexo puede entenderse socialmente como una expectativa de la relación, con posibles repercusiones sobre la autonomía sexual y la salud sexual y emocional de las personas involucradas^(22)^.

La pareja desempeña un papel importante en el apoyo emocional y práctico, aunque no siempre de manera efectiva, lo que puede generar dificultades de comunicación y conflictos. La adaptación a la parentalidad plantea desafíos para la dinámica de pareja y la vida sexual. De este modo, se destaca la importancia del apoyo familiar y profesional durante el ciclo de embarazo y puerperio, con acciones educativas sobre los cambios fisiológicos y emocionales del puerperio y sus impactos en la sexualidad^(23)^.

Además, es importante considerar que el lapso de tiempo transcurrido entre el puerperio y la recolección de datos puede haber influido en las percepciones relatadas por las participantes. Aunque el recuerdo de las experiencias permite la elaboración y la reinterpretación de los acontecimientos vividos, también puede estar sujeto a sesgos de memoria, ya que los relatos retrospectivos implican procesos de reconstrucción e interpretación a lo largo del tiempo, lo que puede dar lugar a una atenuación o intensificación de las experiencias vividas. En este sentido, los relatos pueden reflejar tanto lo vivido en ese momento como interpretaciones elaboradas posteriormente, lo cual debe tenerse en cuenta en el análisis de los hallazgos^(24)^.

### Influencia de los Tipos de Parto en la Experiencia de la Sexualidad Durante el Puerperio

Diversos factores influyen en el tiempo de retorno a la actividad sexual durante el puerperio, como las comorbilidades maternas, el miedo a un nuevo embarazo, el miedo al dolor, el grado de lesión perineal y la lactancia materna^(25)^. Entre las entrevistadas, se observó la percepción de que la vía de parto puede afectar la sexualidad, especialmente debido a laceraciones o episiotomías, que pueden provocar dispareunia, miedo, disminución de la libido y de la lubricación. Además, se mencionaron inseguridades relacionadas con la cicatriz de la cesárea y con los cambios en los genitales.

El parto vaginal puede provocar alteraciones genitales, como traumatismo perineal y dispareunia, aunque presenta beneficios para la salud materna y neonatal, con posibles complicaciones que pueden tratarse con fisioterapia. La cesárea, por su parte, puede generar molestias en la cicatriz y afectar la función sexual, aunque no existe una asociación directa entre el tipo de parto y la función sexual^(26)^. Se ha demostrado que las personas que se someten a una cesárea o a un parto vaginal quirúrgico con laceración o episiotomía pueden tardar más tiempo en retomar la actividad sexual, siendo la presencia de una pareja estable un factor que facilita este proceso^(27)^.

En este contexto, se destaca la importancia del movimiento de humanización del parto, que busca reducir la medicalización y la violencia obstétrica, promoviendo el protagonismo de las mujeres y el reconocimiento de sus derechos. En el ámbito de la sexualidad, intervenciones como la episiotomía pueden provocar trauma perineal, dispareunia y posibles disfunciones sexuales, mientras que las laceraciones pueden ocurrir de manera fisiológica durante el trabajo de parto. Ambas pueden prevenirse o minimizarse con fisioterapia pélvica y técnicas de estiramiento perineal, aunque estas prácticas no se promueven lo suficiente, a pesar de su carácter mínimamente invasivo^(28)^.

Los relatos también pusieron de manifiesto la insatisfacción con la imagen corporal después del parto, relacionada tanto con los cambios derivados del parto vaginal como con la cicatriz de la cesárea. Los cambios estéticos corporales, aunque esperados, pueden generar frustración y afectar la autoimagen y la vida sexual de las personas que han vivido el puerperio^(29)^. Así, se observa que las diferentes vías de parto pueden influir en la experiencia sexual durante el puerperio, lo que refuerza la importancia de la orientación y los espacios de diálogo sobre el tema, así como del acceso a información de calidad sobre el cuerpo, la sexualidad y los cuidados en este contexto.

En este sentido, la autoimagen corporal es un importante determinante psicológico de la sexualidad, pudiendo preceder e influir en el deseo sexual. Evidencias recientes indican que la percepción negativa del propio cuerpo en el posparto está asociada a una reducción de la autoestima, a una disminución de la satisfacción sexual y a una mayor vulnerabilidad emocional, lo que refuerza la necesidad de enfoques que consideren la subjetividad femenina en este período^(30)^.

### Orientaciones Sobre la Sexualidad en el Puerperio

El enfoque de las consultas posparto sigue centrado en la concepción, con escasa inclusión del diálogo con las parejas^(31)^. Teniendo en cuenta los cinco parámetros de salud del individuo, resulta esencial abordar la sexualidad y su impacto en la calidad de vida de la puérpera y de la pareja^(32)^. Las entrevistas ponen de manifiesto la falta de información profesional que valide como normales las experiencias vividas en este período, lo que refuerza la necesidad de una orientación calificada.

Además, es importante considerar que el perfil de las participantes, en su mayoría mujeres blancas y con un mayor nivel educativo, puede influir en el acceso a la información y a la atención de salud. La literatura señala que los marcadores sociales, como la raza, la clase social y el nivel educativo, entendidos desde la perspectiva de la interseccionalidad, afectan el acceso a los servicios de salud y la calidad de la atención recibida, y las mujeres en contextos de mayor vulnerabilidad pueden enfrentar barreras aún más significativas para acceder a una orientación calificada^(33)^.

Dada la influencia de Internet en la comunicación y la educación en salud, este medio se utiliza con frecuencia para aclarar dudas. Sin embargo, la información obtenida puede generar inseguridad y afectar negativamente la experiencia de la sexualidad. Las cuestiones relacionadas con la actividad sexual aún suscitan dudas entre las mujeres en el posparto, asociadas a la vergüenza y al temor de abordar el tema con los profesionales de la salud^(34)^.

Se reconoce la importancia de brindar información a la pareja y capacitar a los profesionales para abordar la sexualidad durante el puerperio. En Brasil, el Ministerio de Salud cuenta con los Cuadernos de Atención Básica en Salud Sexual y Reproductiva, que orientan las prácticas calificadas en este campo^(35)^. El método *Permission, Limited Information, Specific Suggestions, Intensive Therapy* se destaca como un modelo de consulta que el personal de enfermería puede aplicar en el manejo de las disfunciones sexuales. Las recomendaciones hacen hincapié en tomar en cuenta el contexto sociocultural, brindar apoyo emocional y psicológico, y adoptar enfoques no farmacológicos, junto con una educación sexual de calidad. Además, el asesoramiento debe comenzar en la etapa prenatal, identificando disfunciones y orientando sobre los cambios fisiológicos para preparar a la pareja para el ciclo de embarazo y puerperio^(36)^.

Se observa una falta de atención por parte de los profesionales de la salud, incluidos los enfermeros, al abordar la sexualidad, lo que se refleja en la escasez de estudios sobre intervenciones terapéuticas. La discusión del tema puede favorecer una comunicación más abierta entre las mujeres embarazadas, las puérperas y los profesionales de la salud, además de estimular futuras investigaciones. Por ello, es esencial capacitar especialmente a los enfermeros, ya que la sexualidad es un pilar de la atención en salud. El equipo debe actuar de manera eficaz desde la etapa prenatal, tomando en cuenta las particularidades de cada persona, para superar las dificultades relacionadas con la sexualidad^(37)^.

Aunque el número de participantes es adecuado para la propuesta del estudio, no permite hacer generalizaciones, dada la magnitud de la población investigada. Cabe destacar, sin embargo, que la investigación cualitativa busca comprender el fenómeno a partir de las narrativas y singularidades de las participantes. Por lo tanto, se sugiere realizar nuevos estudios con estrategias de búsqueda activa y una mayor difusión, combinadas con el método de bola de nieve, para lograr una mayor representatividad de las puérperas.

Entre las limitaciones del estudio, se destaca, en primer lugar, el intervalo de tiempo entre la experiencia del puerperio, la recolección de datos (2021-2022) y el período de publicación, lo que pudo haber influido en los relatos a partir de procesos de rememoración, elaboración y resignificación de las experiencias vividas. Aunque la memoria permite reflexiones más maduras sobre lo vivido, los relatos retrospectivos están sujetos a sesgos, lo que puede atenuar o intensificar ciertas percepciones.

Otra limitación se refiere al perfil de las participantes, compuesto en su mayoría por mujeres blancas, con un mayor nivel educativo y residentes en un contexto urbano. Estos marcadores sociales de raza, clase y género pueden influir en el acceso a la información, a los servicios de salud y a las posibilidades de atención, lo que limita la extrapolación de los resultados a mujeres en contextos de mayor vulnerabilidad social, racial y económica.

Además, aunque el número de 37 participantes permitió identificar recurrencias temáticas y alcanzar la saturación de los datos, se trata de un contingente elevado para estudios cualitativos que exigen una mayor densidad analítica en cuanto a la profundidad individual, lo que puede haber limitado un análisis más minucioso de cada narrativa. Esta limitación se ve acentuada por el uso combinado de entrevistas dialógicas y cuestionarios autoadministrados, ya que este último formato restringe la posibilidad de explorar en profundidad los significados emergentes en comparación con la entrevista sincrónica.

Como contribución a la enfermería, se espera que el estudio fomente el perfeccionamiento profesional y amplíe el enfoque de la sexualidad femenina, desde el período prenatal hasta el puerperio, estimulando el diálogo sobre el tema en los diferentes ámbitos de actuación del personal de enfermería. Además, los resultados pueden servir de base para nuevas investigaciones y contribuir a la mejora de las acciones de educación continua en salud.

## CONCLUSIÓN

La investigación abarcó la experiencia de la sexualidad en el puerperio, poniendo de manifiesto que, para muchas personas, ejercer la sexualidad de manera satisfactoria en este período constituye un desafío. Aunque reconocen su importancia para la salud, la autoestima y el mantenimiento de relaciones saludables, las exigencias de la parentalidad a menudo reducen el tiempo dedicado a la intimidad, lo que hace que el diálogo entre la pareja sea fundamental para preservar el vínculo.

Se señalaron factores biológicos, como el dolor durante las relaciones, la disminución de la libido, los efectos de la lactancia materna y la insatisfacción con el cuerpo, como limitaciones para volver al patrón sexual anterior al embarazo. Además, tanto el parto vaginal como la cesárea se asociaron con molestias que pueden afectar la sexualidad. También se puso de manifiesto una falta de sensibilidad en el enfoque de la sexualidad por parte de los profesionales de la salud durante el ciclo de embarazo y puerperio, con un predominio de orientaciones limitadas a los métodos anticonceptivos y al período de reposo.

El estudio refuerza la necesidad de ampliar el enfoque de la sexualidad en la atención de la salud, especialmente en enfermería, desde la formación académica hasta la práctica asistencial, promoviendo la educación en salud y el apoyo calificado a las personas que atraviesan el puerperio.

## Data Availability

Todo el conjunto de datos en el que se basan los resultados de este estudio se ha publicado en: https://repositorio.ufsc.br/handle/123456789/248224.
